# Tropical Plantains, Tubers, and Roots: Advancing Food Quality, Safety, and Processing Innovations for Global Nutritional Equity

**DOI:** 10.1002/fsn3.72497

**Published:** 2026-10-28

**Authors:** Mundéné‐Timothée Junior Lawrence, Veeranna Hitlamani, Njintang Yanou Nicolas, Aashitosh A. Inamdar

**Affiliations:** ^1^ Chemical Engineering Department, Higher Technical Teachers Training College University of Douala Douala Cameroon; ^2^ Flour Milling, Baking & Confectionery Technology Department CSIR‐Central Food Technological Research Institute Mysuru India; ^3^ Department of Biological Sciences, Faculty of Sciences University of Ngaoundere Ngaoundere Cameroon

**Keywords:** bioactive compounds, food safety, functional nutrition, plantains, post‐harvest technology, sustainable processing, tropical tubers

## Abstract

Plantains and tropical tubers and roots (PTRs), predominantly cassava, sweet potato, yam, potato, and taro, constitute indispensable nutritional and caloric pillars for over 500 million people across tropical and subtropical agroecological zones. Beyond their conventional role as energy‐dense carbohydrate matrices, these crops represent strategic reservoirs of dietary fiber, essential vitamins, and bioactive phytonutrients. However, their sustainable integration into high‐value global food chains is significantly constrained by high post‐harvest losses (ranging from 30% to 40%), rapid physiological deterioration, infrastructural deficits, and hazardous antinutritional factors (ANFs), including cyanogenic glycosides in cassava, glycoalkaloids in potato, and acrid calcium oxalate raphides in taro. This review provides an exhaustive synthesis of recent global production dynamics, molecular starch architectures, and emerging functional food applications of PTRs. It evaluates the efficacy of traditional processing (fermentation, soaking, and thermal treatment) versus modern non‐thermal and thermomechanical innovations (high‐pressure processing [HPP], extrusion, and enzymatic modification) in enhancing food safety, digestibility, and techno‐functional properties. Although traditional fermentation remains a cornerstone for detoxification and probiotic enrichment, modern technologies like HPP, pulsed electric fields (PEF), and encapsulation nanotechnology offer superior retention of heat‐sensitive micronutrients and antioxidants. Furthermore, the functional role of resistant starch fractions (RS1–RS5) in modulating metabolic health and the glycemic index is appraised as a critical frontier for functional food development. Ultimately, this work establishes an integrated processing continuum that bridges ancestral bioprocessing with eco‐efficient engineering, international food safety standards (Codex Alimentarius, EFSA), and gender‐responsive socioeconomic empowerment to reduce post‐harvest waste and advance global nutritional equity.

## Graphical abstract

Plantains, tubers, and roots (PTRs) represent strategic nutritional and caloric pillars for over 500 million people across tropical regions. This review evaluates traditional bioprocessing against eco‐innovative thermomechanical and non‐thermal technologies (HPP, extrusion, encapsulation) to overcome severe post‐harvest losses and antinutritional risks. Bridging ancestral detoxification with advanced molecular starch tailoring (RS1–RS5) optimizes micronutrient bioaccessibility, enhances functional food safety, and drives sustainable global nutritional equity.

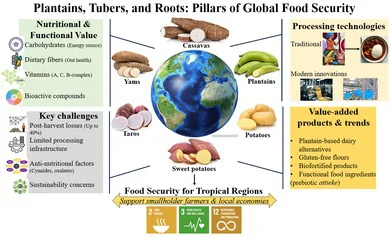

## 1 Introduction

Plantains, tubers, and roots (PTRs) constitute the foundation of food security for over 500 million people across tropical and subtropical regions (Scott 2021; Lebot 2020). Beyond their role as caloric pillars, these crops represent complex matrices rich in dietary fiber, essential vitamins, and minerals (Thiele et al. 2017; Scott 2021). However, within the current global public health framework, interest in these commodities has shifted from mere energy security toward nutritional quality and the promotion of health equity.

Plantains (*Musa paradisiaca*) are distinguished from dessert bananas by their high starch content and requirement for cooking (Dufour et al. 2009) coexisting with a diverse range of underground roots and tubers. Among these, cassava ( *Manihot esculenta* ), yam (*Dioscorea* spp.), potato ( *Solanum tuberosum* ), taro ( *Colocasia esculenta* ), and sweet potato ( *Ipomoea batatas* ) are notable for their unique biochemical properties and energy storage capacities (Alamar et al. 2017; Dufour et al. 2021). Characterizing these quality attributes is fundamental to optimizing industrial processing and ensuring their nutritional functionality.

Global production of PTRs is steadily increasing (Figure 1), bolstered by their exceptional resilience to varying agroecological conditions (Ritchie et al. 2023). In Sub‐Saharan Africa and Latin America, these crops energize local food systems (Adesokan et al. 2023; Nainggolan et al. 2024). Nevertheless, the sector faces critical challenges: high post‐harvest losses and the risk of microbiological or chemical contamination during inadequate storage. Innovation in preservation technologies and food safety monitoring is therefore imperative to reduce waste and add value to these resources.

**FIGURE 1:**
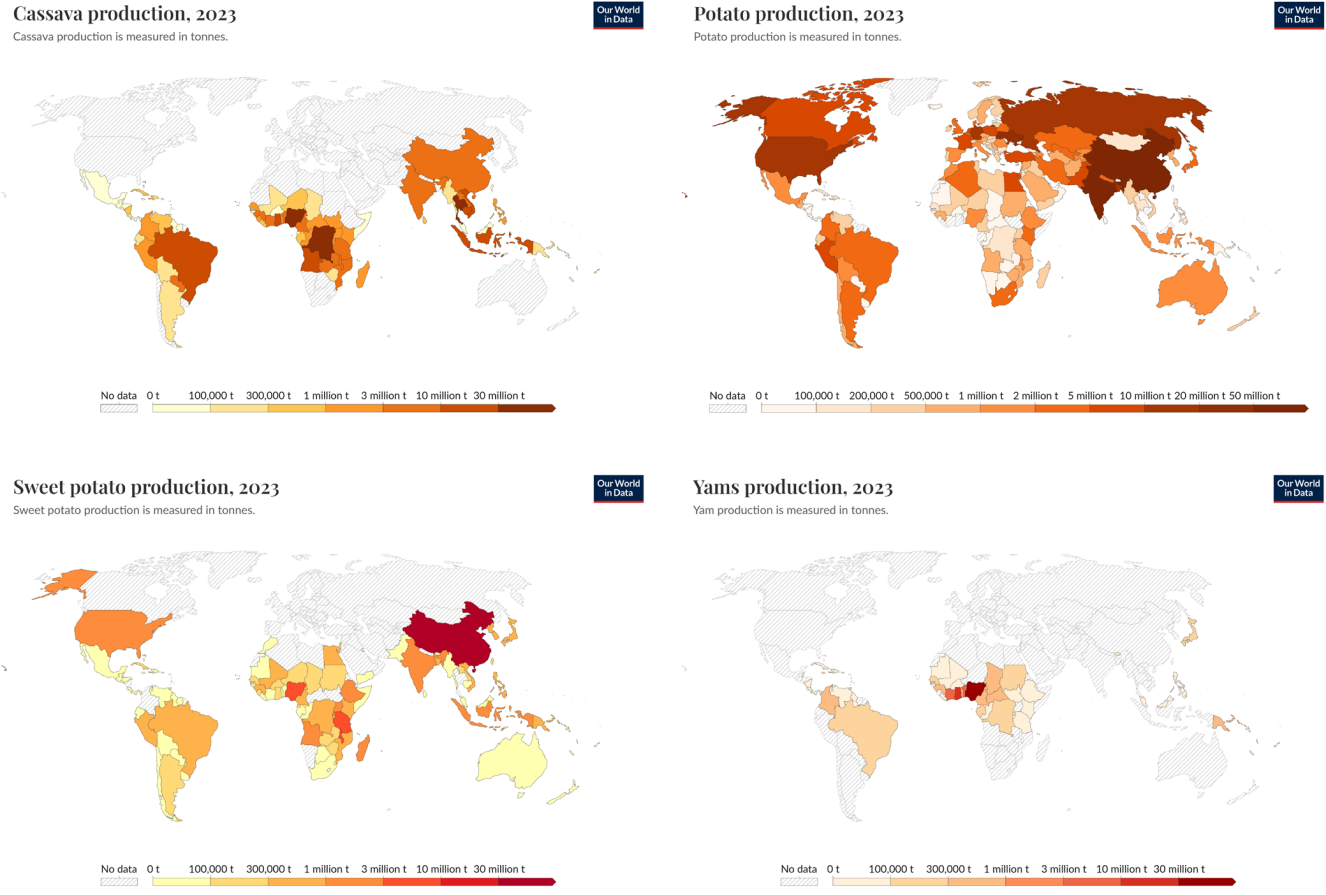
Breakdown of the 2023 global agricultural production of major root and tuber crops (Ritchie et al. 2023).

The evolution of traditional food systems toward modern applications requires a synergy between ancestral knowledge and food engineering. Although artisanal methods, such as cassava fermentation for *gari* or *attiéké* and plantain frying, persist (Kuku‐Shittu et al. 2013; Arora et al. 2022), new technological trajectories are emerging. These include the development of low‐glycemic index gluten‐free flours, the extraction of beneficial phytochemicals, and the enhancement of nutrient bioavailability through minimal processing techniques (Adi 2024). Such innovations aim not only to extend shelf life but also to guarantee rigorous sensory and safety standards.

Given the socioeconomic and health significance of PTRs, their sustainable integration into global food systems relies on simultaneous advances in processing technologies, food safety practices, and the understanding of their nutraceutical properties. This review provides a comprehensive examination of the nutritional qualities, processing innovations, and food safety challenges associated with tropical roots, tubers, and plantains. By synthesizing knowledge on health benefits and potential risks (e.g., toxicology, mycotoxins), this work highlights the vital role of these crops in combating diet‐related diseases and promoting sustainable, equitable food systems.

## 2 Literature Search Strategy and Selection Framework

To ensure methodological rigor, transparency, and comprehensive coverage of the multidisciplinary evidence base, literature retrieval for this review was executed using a structured narrative review framework informed by PRISMA 2020 search principles. Comprehensive bibliographic searches were conducted across five major electronic databases: Web of Science (Core Collection), Scopus, PubMed/MEDLINE, ScienceDirect, and Google Scholar, covering peer‐reviewed literature published from January 2000 through December 2025. The Boolean search algorithms integrated crop‐specific taxonomic designations with functional, chemical, and processing keywords: (“plantain” OR “*Musa paradisiaca*” OR “cooking banana” OR “cassava” OR “ *Manihot esculenta* ” OR “yam” OR “Dioscorea” OR “sweet potato” OR “ *Ipomoea batatas* ” OR “taro” OR “ *Colocasia esculenta* ”) AND (“nutritional quality” OR “starch architecture” OR “resistant starch” OR “antinutritional factors” OR “cyanogenic glycosides” OR “glycoalkaloids” OR “oxalates” OR “detoxification kinetics” OR “food safety” OR “processing innovations” OR “high‐pressure processing” OR “pulsed electric fields” OR “extrusion” OR “nanoencapsulation” OR “post‐harvest losses”). The search was supplemented by cross‐referencing normative technical documents from international bodies, including the Food and Agriculture Organization (FAO), World Health Organization (WHO), Codex Alimentarius Commission, and the European Food Safety Authority (EFSA).

Inclusion criteria comprised: (i) primary peer‐reviewed experimental studies reporting validated quantitative or qualitative data on proximate composition, bioactive profiling, starch rheology, or processing impacts of tropical PTRs; (ii) authoritative scientific reviews and meta‐analyses on starch biopolymers and modern non‐thermal preservation technologies; and (iii) official regulatory and policy standards on food safety thresholds. Exclusion criteria eliminated: (i) studies lacking peer review or methodological validation; (ii) agricultural agronomy trials with no reported food, processing, or nutritional characterization; and (iii) studies focusing on temperate forage plantain species ( *Plantago lanceolata* ). From an initial identification of 1842 records, title and abstract screening followed by full‐text critical appraisal yielded a final corpus of 172 high‐impact studies synthesized in this review.

## 3 Global Production Trends of Roots, Tubers, and Plantains

The strategic importance of plantains, tubers, and roots (PTRs) in ensuring food security and nutritional equity across tropical and subtropical regions cannot be overstated (Saranraj et al. 2019; Chovatiya et al. 2025). Between 2020 and 2023, global production of these commodities demonstrated significant upward momentum (Table 1), reflecting their vital role as resilient carbohydrate sources in the face of shifting climate patterns and global supply chain disruptions (Ritchie et al. 2023). This growth is not merely quantitative; it underscores a fundamental shift toward the industrial valorization of these crops, moving them from subsistence staples to essential raw materials for the global bio‐economy.

**TABLE 1:** Trends in the production of roots, tubers, and plantains (2020 and 2023).

Crop	Year	Global production (MT)	Top producing region (MT)	Leading country (MT)
Cassava	2020	310,284,506	Africa: 194,803,280	Nigeria: 55,565,610
	2023	335,342,304	Africa: 213,079,550	Nigeria: 62,690,092
Potatoes	2020	369,091,672	Asia: 187,700,780	China: 92,852,720
	2023	383,337,349	Asia: 204,418,060	China: 93,491,816
Yam	2020	81,809,507	Africa: 79,836,616	Nigeria: 56,345,530
	2023	89,345,130	Africa: 87,644,730	Nigeria: 61,921,740
Sweet potato	2020	92,095,711	Asia: 58,650,376	China: 51,786,510
	2023	93,948,148	Asia: 58,189,240	China: 51,629,136
Taro	2020	17,769,410	Africa: 14,850,575	Nigeria: 7,867,170
	2023	18,073,943	Africa: 15,143,257	Nigeria: 8,334,557
Plantain	2020	43,822,383	Africa: 30,842,646	Uganda: 11,756,441
	2023	46,885,407	Africa: 30,681,485	Uganda: 11,090,315

### 3.1 Cassava: A Pillar of Food Security and Industrial Feedstock

Cassava ( *M. esculenta* ) remains a cornerstone of nutritional resilience, particularly in sub‐Saharan Africa. Global production rose from 310.2 million metric tons (MT) in 2020 to approximately 335.3 million MT in 2023 (Table 1). Africa's dominance is clear, with production increasing from 194.8 to 213.1 million MT, led by Nigeria (62.7 million MT) and the Democratic Republic of Congo (45.1 million MT). Beyond its role in public health, cassava is increasingly viewed through the lens of food quality and processing innovation. Its high starch content makes it a competitive raw material for gluten‐free flours and industrial applications. However, maximizing its potential requires addressing biochemical challenges, such as rapid post‐harvest physiological deterioration (PPD) and the management of cyanogenic glucosides to ensure consumer safety (Odeku 2013).

### 3.2 Plantains: Regional Stability and Market Diversification

Production of plantains and cooking bananas (*M. paradisiaca*) has remained relatively stable in Africa (~30.6 million MT), yet it shows an intriguing geographic expansion in Asia and the Caribbean (Ritchie et al. 2023). In countries like Uganda and Cameroon, plantains are essential to the dietary diversity of over 70 million people (Dury et al. 2002; Mundéné‐Timothée et al. 2024). From a food safety and quality perspective, the stability of this sector relies on improving traditional processing techniques, such as frying and drying, to minimize the formation of acrylamides and ensure the retention of essential micronutrients during value‐added production.

### 3.3 Potato: Globalization and Quality Control

Among tubers, the potato ( *S. tuberosum* ) exhibits the most robust global footprint, with production exceeding 383 million MT in 2023. The significant leadership of China (93.5 million MT) and India (60.1 million MT) highlights the crop's strategic shift toward the Global South. As a highly perishable commodity, the potato industry faces rigorous quality monitoring requirements, particularly regarding storage temperatures and the prevention of glycoalkaloid accumulation (Platt 2008). Innovations in minimal processing technologies are currently being deployed to maintain the sensory and nutritional attributes of fresh‐cut potato products in expanding urban markets (Maniriho et al. 2021).

### 3.4 Sweet Potato: Resilience and Biofortification

The sweet potato ( *I. batatas* ) serves as a critical tool for combating “hidden hunger” due to its rich phytochemical profile, including beta‐carotene in orange‐fleshed varieties. Global production reached 93.9 million MT in 2023. Although China remains the dominant producer, significant growth in African nations like Malawi (8.0 million MT) reflects a concerted effort toward dietary diversification (Odeku 2013). The primary challenge for this crop lies in post‐harvest handling and the development of sustainable food systems that can preserve its delicate antioxidant compounds during processing.

### 3.5 Taro and Yam: Traditional Knowledge Meets Modern Supply Chains

Taro ( *C. esculenta* ) and yams (*Dioscorea* spp.) represent crops deeply embedded in the cultural heritage of Africa. Yam production, in particular, saw a notable expansion from 81.8 to 89.3 million MT, driven almost entirely by West African nations like Nigeria and Ghana (Table 1). Taro, though smaller in volume (~18 million MT), remains vital for localized food security in Nigeria and Cameroon. For both crops, the path to industrial production requires overcoming bottlenecks in mechanization and the standardization of quality attributes for traditional products like *achu* (pounded Taro) and *fufu* (pounded yam).

In conclusion, the rising production trends of PTRs necessitate a multisectoral approach that prioritizes food quality assurance and safety monitoring. To fully unlock the economic and nutritional potential of these tropical crops, the integration of innovative processing technologies, such as improved fermentation systems and energy‐efficient drying, is essential for reducing post‐harvest losses and ensuring the delivery of safe, value‐added food products to a growing global population.

## 4 Nutritional Composition and Health Benefits of PTRs

Plantains, tubers, and roots (PTRs) serve as indispensable matrices of macronutrients, micronutrients, and bioactive compounds. Their nutritional architecture is highly dynamic, influenced by genotypic variation, agro‐climatic conditions, and post‐harvest handling (Oke and FAO 1990; Chandrasekara and Kumar 2016; Lebot 2020). Although traditionally valued for their energy density, contemporary food science emphasizes their role in nutritional equity and the prevention of non‐communicable diseases through their unique phytochemical profiles.

### 4.1 Macro‐ and Micronutrient Profiles

PTRs are characterized by high carbohydrate concentrations, primarily in the form of starch, ranging from 20% to 35% on a fresh‐weight basis (Table 2). Cassava and sweet potatoes are notably energy‐dense, with starch comprising up to 90% of their dry matter (Scott 2021; Atsa'am and Wario 2022). A critical quality attribute of PTRs is their dietary fiber content (2%–4%), which modulates lipid metabolism and attenuates postprandial glycemic responses (Alam et al. 2016). Unripe plantains are particularly significant in this regard due to their high resistant starch (RS) levels, which function as prebiotics (Oluwajuyitan and Ijarotimi 2019). Although protein (1%–3%) and fat (< 1%) contents are naturally low, the quality of these proteins, such as sporamine in sweet potatoes, adds functional value beyond basic nutrition.

**TABLE 2:** Nutritional and proximate composition of selected PTR crops (raw material per 100 g).

Nutrients (per 100 g)		Crops (raw material)						
		Cassava	Plantain	Potatoes		Sweet potatoes	Taro	Yam
				White flesh	Red flesh			
Macronutrients								
Energy (kcal)		160.0	122.0	69.0	70.0	86.0	112.0	118.0
Carbohydrates (g)		38.1	31.9	15.7	15.9	20.1	26.5	27.9
Dietary fiber (g)		1.8	2.3	2.4	1.7	3.0	4.1	4.1
Protein (g)		1.4	1.3	1.7	1.9	1.6	1.5	1.5
Fat (g)		0.3	0.4	0.1	0.1	0.1	0.2	0.2
Total sugars (g)		1.7		1.2	1.3	4.2		0.5
Minerals								
Electrolytes	Sodium (mg)	14.0	4.0	16.0	18.0	55.0	11.0	9.0
	Potassium (mg)	271.0	499.0	407.0	455.0	337.0	591.0	816.0
Magnesium (mg)		21.0	37.0	21.0	22.0	25.0	33.0	21.0
Calcium (mg)		16.0	3.0	9.0	10.0	30.0	43.0	17.0
Phosphorus (mg)		27.0	34.0	62.0	61.0	47.0	56.0	55.0
Iron (mg)		0.9	0.6	0.7	0.8	0.9	0.6	1.0
Zinc (mg)			0.14				0.23	
Vitamins								
Ascorbic acid (C) (mg)		20.6	18.4	19.7	8.6	2.4	4.5	17.1
Thiamine (B1) (mg)		0.09	0.052	0.07	0.08	0.08	0.09	0.11
Riboflavin (B2) (mg)		0.05	0.054	0.03	0.03	0.06	0.025	0.03
Niacin (B3) (mg)		0.85	0.69	1.07	1.15	0.56	0.6	0.55
Pyridoxine (B6) (mg)		0.09	0.3	0.203	0.17	0.21	0.3	0.3
Folate (B9) (μg‐DFE)		27.0	22.0	18.0	18.0	11.0	22.0	23.0
Vitamin E (mg)		0.19	0.14	0.01	0.01	0.26	2.4	0.35
Vitamin K (μg)		1.9	0.7	1.6	2.9	1.8	1.0	2.3
Vitamin A (IU)		13.0	948.37	8.0	7.0	14,187	76.0	138
Bioactive compounds								
Resistant starch (g)		1.5 (cooked & cooled)	2.1	3.2 (cooked & cooled)		1.4	2.3	2.0
Antioxidants		Low	Phenolic acids (p‐coumaric acid) Quercetin/Quercetagetin Flavones/2″‐O‐xylosylvitexin	Chlorogenic acid		High anthocyanins Β‐carotene	Anthocyanins	Diogenin (a phytoestrogen)
Anti‐nutrients		Cyanogenic glycosides	Low	Solanine (glycoalkaloids)		Low	Oxalates	Oxalates
Health benefits		Gluten‐free alternative for celiac disease Energy‐dense for active populations	Prebiotic fiber promotes gut health Rich in electrolytes (potassium/magnesium)	Good vitamin C source (boosts immunity) Resistant starch, when cooled, aids gut health		Vision and immune support (vitamin A) Antioxidants reduce inflammation	Low‐calorie, high‐fiber option for weight management Antioxidant‐rich purple varieties	Supports blood pressure regulation Potential hormonal balance benefits (diosgenin)
Potential concerns		Cyanogenic glycosides (must be processed to avoid toxicity) Low micronutrient density	High glycemic load when ripe (less so when cooked green)	Glycoalkaloids (solanine) in green skins may be toxic High glycemic index when fried/mashed		Higher sugar content (4.2 g) may affect blood glucose if consumed excessively	Oxalates may inhibit calcium absorption (risk for kidney stones)	Oxalates may contribute to kidney stones in susceptible individuals

From a micronutrient perspective, PTRs are vital for combating “hidden hunger.” Orange‐fleshed sweet potatoes are a superior source of β‐carotene (precursor to Vitamin A), providing approximately 709 μg RAE/100 g (Newilah et al. 2005; Neela and Fanta 2019; Uzoaga et al. 2020). Conversely, yams and taro excel in mineral density, particularly potassium (up to 816 mg/100 g) and magnesium, which are essential for cardiovascular and neuromuscular homeostasis (Alamar et al. 2017). Although anti‐nutrients like phytic acid can sequester minerals, processing innovations such as fermentation and controlled steaming significantly enhance mineral bioavailability (Kuku‐Shittu et al. 2013).

### 4.2 Bioactive Compounds and Functional Health Properties

The integration of PTRs into the global diet is increasingly supported by their complex profiles of secondary metabolites, which include phenolic acids, anthocyanins, saponins, and functional proteins (Table 2). Beyond providing basic sustenance, these crops function as delivery vehicles for bioactive compounds that modulate physiological pathways associated with chronic disease prevention and health promotion (Chandrasekara and Kumar 2016; Chovatiya et al. 2025).

#### 4.2.1 Profiling of Major Bioactive Secondary Metabolites

Plantain and major tubers, including potato, sweet potato, yam, taro, and cassava, possess a sophisticated array of bioactive secondary metabolites. Key among these are phenolic acids, such as chlorogenic and *p*‐coumaric acids, and anthocyanins, which provide the vibrant pigmentation in purple and red cultivars. Furthermore, these matrices contain specialized saponins (e.g., diosgenin) and unique functional storage proteins, namely dioscorin in yams and sporamine in sweet potatoes. Although essential micronutrients like carotenoids (β‐carotene, lutein) and ascorbic acid are prevalent, these crops also contain anti‐nutritional factors such as tannins and phytates. Scientific rigor in food processing is essential, as these factors are typically mitigated through soaking, fermentation, or thermal treatments to unlock the full functional potential of the crop (Oladele et al. 2023; Udomkun et al. 2021; Oyeyinka and Afolayan 2019; Chovatiya et al. 2025).

#### 4.2.2 Antioxidant and Immunomodulatory Dynamics

PTRs serve as critical exogenous sources of antioxidants, which are vital for neutralizing reactive oxygen species (ROS) and mitigating oxidative stress‐related pathologies. Pigmented varieties, particularly purple yams and red‐fleshed potatoes, demonstrate superior radical scavenging activities. These effects are mediated through the up‐regulation of hepatic antioxidant enzymes, including superoxide dismutase (SOD) and glutathione peroxidase (Han et al. 2007; Chan et al. 2010). Beyond antioxidant capacity, the immunomodulatory potential of yams is largely attributed to dioscorin. This storage protein has been shown to enhance innate immunity by stimulating macrophage activity and modulating cytokine synthesis (Liu et al. 2007). Clinical evidence further suggests that regular intake of these pigmented cultivars correlates with a systemic reduction in pro‐inflammatory markers, such as C‐reactive protein (CRP), thereby promoting a balanced inflammatory response (Chandrasekara and Kumar 2016).

#### 4.2.3 Chemopreventive and Cytotoxic Potential

The chemopreventive potential of PTR bioactive extracts is characterized by their ability to intervene during the early stages of carcinogenesis. Anthocyanins derived from purple sweet potatoes have been shown to inhibit carcinogen‐induced mutagenesis. Simultaneously, specific potato glycoalkaloids, such as α‐chaconine and α‐solanine, along with yam‐derived saponins, exhibit potent antiproliferative effects. These compounds induce apoptosis in liver and colon cancer cell lines by modulating the NF‐κB signaling pathway, a central regulator of inflammation and cell survival (Lee et al. 2004; Auyeung et al. 2009). It is noteworthy, however, that the concentration and efficacy of these polyphenols are highly sensitive to agronomic variables and post‐harvest storage conditions, emphasizing the need for standardized quality control in the supply chain (Thompson et al. 2009).

#### 4.2.4 Metabolic Regulation: Glycemic and Lipidemic Control

PTRs play a transformative role in the management of metabolic syndrome through hypoglycemic, hypocholesterolemic, and anti‐obesity mechanisms. Clinical studies on the “Caiapo” sweet potato variety have demonstrated significant reductions in fasting blood glucose and improved insulin sensitivity in patients with type 2 diabetes (Kusano and Abe 2000). In the context of lipid management, diosgenin from yams serves as a potent agent that inhibits intestinal cholesterol absorption by targeting the NPC1L1 transport protein while simultaneously promoting biliary excretion (Xiong et al. 2009). Furthermore, the synergy between anthocyanins and sporamine in sweet potatoes exerts anti‐obesity effects by activating the pathway, which inhibits adipocyte differentiation and lipid accumulation (Hwang et al. 2011). These findings position PTRs not merely as staples but as essential components of therapeutic nutrition and sustainable food systems.

### 4.3 Comparative Analysis: PTRs Versus Cereals

The nutritional profiles of plant‐based tuberous resources (PTRs) diverge significantly from those of conventional cereals, with profound implications for food quality, metabolic health, and dietary diversity (Table 3). Although cereals such as wheat, rice, and maize are the primary drivers of global caloric intake due to their higher protein content (7%–15%) and storage stability, PTRs offer distinct advantages in terms of carbohydrate digestibility, micronutrient bioavailability, and functional bioactive potential (Nuwamanya et al. 2011; Chovatiya et al. 2025).

**TABLE 3:** Comparative nutritional and functional profile: PTRs versus conventional cereals.

Characteristics		Tubers & plantains	Cereals
Examples		Cassava, sweet potato, yam, taro, potato, plantain	Wheat, corn, millet
Energy value		High (especially cassava/plantain). A significant source of complex carbohydrates	High. Dominant complex carbohydrates (starch) for sustained energy
Primary nutritional composition		Carbohydrates: Rich in complex carbohydrates (starch), some with resistant starch (plantain, cooked then cooled potato) Fiber: Moderate, variable content (varies by species), generally good sources (soluble & insoluble, especially sweet potato, taro, plantain) Proteins: Low content (1%–3%), limited quality (deficient in essential AAs like lysine) Fats: Very low content (< 1%) Vitamins: Vitamin C (potato, plantain), A/β‐carotene (orange sweet potato), B6, folate (yam/taro) Minerals: Potassium (+++), magnesium, and manganese. Antinutrients reduce bioavailability	Carbohydrates: Rich in complex carbohydrates (starch) Fiber: Moderate (++ whole wheat/millet), varies by type (whole vs. refined grains). Soluble (β‐glucans) and insoluble Proteins: Moderate (7%–15%), better quality (corn/millet deficient in lysine/tryptophan; wheat in lysine). Some contain gluten (wheat) Fats: Low (except corn: 3%–4%) Vitamins: B Vitamins (niacin, thiamine, riboflavin), Folate (especially whole grains), E (corn), no native C/A Minerals: Iron, zinc, magnesium, selenium. Bioavailability is affected by phytates
Health impacts	+	Digestion: Gentle fibers (cassava) are suitable for intestinal transit. Resistant starch benefits gut flora (plantain, specific potato preparations) Glycemic index: Often moderate (cassava, plantain) compared to some refined starches. Resistant starch helps modulate blood sugar Gluten‐free: Naturally gluten‐free (cassava, sweet potato, yam, taro, plantain), ideal for intolerant people Antioxidants: Rich in antioxidant compounds (carotenoids in sweet potato, phenols in potato, anthocyanins in purple potatoes) for cell protection Metabolic management: Certain compounds (diosgenin from yams) may help regulate cholesterol Immune support: Vitamins A and C are crucial in supporting the immune system Antioxidant, anti‐inflammatory activities Prebiotic effects (fiber, resistant starch)	Digestion: Dietary fibers promote healthy transit Heart health: Fiber (whole grains) can help reduce cholesterol and cardiovascular risks Nutrient‐rich: Provides a wide range of essential B vitamins and minerals Chronic disease prevention: Regular consumption of whole grains is associated with a reduced risk of cardiovascular disease, type 2 diabetes, and certain types of cancer
	−	Toxicological risks (cyanides in raw cassava, glycoalkaloids) Mineral deficiencies (antinutrients) High glycemic index (cooked potato/ripe plantain)	Intolerances (wheat gluten) Mineral deficiencies (phytates) Allergic reactions (wheat/corn proteins) High glycemic index (refined flours)
Bioactive compounds		Diverse: Carotenoids (sweet potato), anthocyanins (purple sweet potato), glycoalkaloids (potato), saponins (yam), chlorogenic acid	Mainly: Phenolic compounds (ferulic acid, vanillic acid), alkylresorcinols (whole wheat), lignans (flax), zeaxanthin (corn)
Antinutrients/precautions		Contain antinutrients (e.g., cyanogens in cassava) requiring adequate cooking and processing to be inactivated Some can be irritating when raw (oxalates in taro) Tannins, phytates, lectins (raw cassava) Glycemic index can vary greatly depending on preparation	Some contain gluten (wheat, barley, rye), an issue for people with celiac disease or gluten sensitivity It may contain phytic acid, which can reduce the absorption of certain minerals (soaking, sprouting, and fermentation can mitigate this), such as lectins (wheat) Some corn varieties may be genetically modified
Culinary applications		Staple Foods: Used as primary starches in many global cuisines (*fufu*, *attiéké*, French fries, purees, stews, soups) Flours/semolina: Cassava (flour, gari, tapioca), sweet potato flour for gluten‐free baking, thickeners Snacks/desserts: Plantain chips, sweet potato cakes, yam‐based desserts Beverages: Fermentation of some tubers for traditional drinks	Breads & baked goods: Wheat flour for breads, cakes, and pasta Staple foods: Rice, pasta, couscous, polenta (corn), porridges (millet) Beverages: Beers (barley, wheat), fermented drinks Processed foods: Biscuits, breakfast cereals, extruded products Animal feed: Major components of animal feed (corn, wheat)
Specific considerations		Cassava: Very energy‐dense, requires rigorous processing to eliminate cyanogens Sweet potato: Rich in beta‐carotene, sweeter than a potato Yam: Source of diosgenin, used in some regions for its medicinal properties Taro: Gentle on digestion once cooked, can be mucilaginous Potato: Source of potassium and vitamin C; resistant starch forms after cooking and cooling Plantain: Fruit eaten as a vegetable, very high in starch, and a good source of potassium	Wheat: Most widely cultivated cereal, staple of Western diets Corn is widely used globally as an energy source but has less fiber and protein than other whole grains Millet: Ancient grain, gluten‐free, resilient to harsh conditions, and an excellent source of fiber and minerals

#### 4.3.1 Carbohydrate Quality and Glycemic Modulation

A defining characteristic of PTRs is the presence of slowly digestible carbohydrates and elevated levels of resistant starch (RS). These attributes are strongly associated with improved glycemic regulation and positive modulation of gut microbiota (Oluwajuyitan and Ijarotimi 2019; Dufour et al. 2021). For instance, unripe plantains consist of 74%–80% carbohydrates, predominantly starch, accompanied by 2%–4% dietary fiber. This composition contrasts sharply with refined cereals, which are frequently depleted of fiber during the milling process (Sojinu et al. 2021; Ogunlakin et al. 2022). Furthermore, cassava's high‐amylose starch confers a glycemic response advantage over polished rice, positioning tubers as strategic components for managing metabolic disorders (Thiele et al. 2017).

#### 4.3.2 Micronutrient Density and Electrolyte Balance

Nutrient density further differentiates these food groups, particularly regarding heat‐labile and fat‐soluble vitamins. Plantains and orange‐fleshed sweet potatoes provide superior concentrations of vitamins A and C compared to most cereals, which typically require industrial fortification to address natural deficiencies (Newilah et al. 2005). Orange sweet potatoes supply approximately 709 μg RAE/100 g of vitamin A, whereas polished rice contributes negligible amounts. Similarly, taro and yams serve as concentrated reservoirs of potassium (591–816 mg/100 g), significantly surpassing maize (287 mg/100 g). This high potassium‐to‐sodium ratio is vital for maintaining cardiovascular health and electrolyte balance (Alamar et al. 2017; Kamanula et al. 2018). Conversely, whole cereals remain superior sources of B‐complex vitamins, such as niacin and folate, primarily due to the preservation of the bran layer (Sojinu et al. 2021).

#### 4.3.3 Processing Impacts and Synergistic Dietary Models

Processing dynamics play a critical role in shaping the final nutritional value of these staples. Although milling reduces the micronutrient density of cereals, post‐harvest technologies for PTRs can actually enhance their functionality. Fermentation (e.g., cassava‐based *gari*) and retrogradation via cooling–reheating cycles in potatoes increase the resistant starch fraction, thereby enhancing their prebiotic potential (Oluwajuyitan and Ijarotimi 2019).

The primary constraints for PTRs remain their low protein density (1%–3%) and the presence of endogenous anti‐nutritional factors (e.g., cyanogens in cassava or glycoalkaloids in potatoes), which necessitate rigorous safety protocols during processing. In contrast, cereals face challenges related to gluten‐associated intolerances and phytate‐induced reductions in mineral bioavailability. Consequently, a “complementary dietary model” grounded in established principles of amino acid complementarity and composite flour technology (Bressani 1993; FAO/WHO 2007; Udomkun et al. 2022; Adegunwa et al. 2024) is scientifically robust: integrating PTR flours into cereal‐based formulations can improve the fiber profile and glycemic index of traditional foods while mitigating the protein paucity of tubers (Adegunwa et al. 2024).

The distinct nutritional profiles of plantains, tubers, and roots (PTRs), from the mineral‐dense matrices of yams to the antioxidant‐rich cultivars of pigmented sweet potatoes, position these crops as critical instruments for mitigating global malnutrition. Unlike conventional monocultures, the biochemical diversity of PTRs allows for the development of composite food systems. For instance, incorporating cassava or unripe plantain flours into wheat‐based formulations serves as a scientifically robust strategy to modulate dietary glycemic indices while partially offsetting the protein‐energy deficits often associated with starchy staples (Adegunwa et al. 2024).

Current research into composite functional foods, including synergistic blends of unripe plantain with bioactive spices, demonstrates how region‐specific food engineering can maximize the bioavailability of essential nutrients and phytochemicals. However, the successful implementation of these nutritional strategies depends heavily on the stability of these compounds during handling. This necessitates a detailed examination of post‐harvest technologies and processing innovations, which are vital for preserving food safety and maintaining the structural integrity of bioactive metabolites throughout the value chain.

## 5 Functional Properties of PTR Starch

Starchy root and tuber crops, predominantly cultivated in tropical and subtropical regions, represent a crucial global energy reservoir due to their high starch yields. Beyond their primary role as dietary staples, these starches serve as multifunctional industrial agents, acting as fillers, glidants, thickeners, binders, and gelling agents in both food and pharmaceutical sectors (Huang 2009; Odeku 2013). The industrial and culinary utility of starch derived from PTRs is not merely a function of its presence but a direct consequence of its granular architecture. The multifaceted interaction between granule morphology, size distribution, and internal crystalline arrangement dictates the thermal and rheological transitions that define food quality and safety.

### 5.1 Starch Physicochemical Characteristics and Resistant Starch (RS) Dynamics

The starch profile of root, tuber, and plantain species exhibits significant interspecific and intraspecific variability, particularly in the amylose/amylopectin ratio, gelatinization temperatures, and granule morphology. These parameters are the primary determinants of the starch's digestibility and its classification into different resistant starch (RS) fractions (Zang et al. 2024).

As illustrated in Figure 2, the formation of RS is a complex interplay between the native botanical structure and processing interventions. RS1 is a physically inaccessible starch trapped within cellular matrices, whereas RS2 consists of native B‐type or C‐type granules that resist enzymatic hydrolysis due to their crystalline packing. It is found in large quantities in green plantains, cassava, yams, and sweet potatoes. Its formation is maximized when tubers and plantains are immature or raw (Remya and Jyothi 2015). RS3 (retrograded starch) is formed during cooling after gelatinization, a process highly dependent on the molecular weight and amylose content of the specific PTR. The content in food can be improved by repeated cycles of heating/cooling. Although rare in nature, RS4 is produced through chemical modifications, including cross‐linking, esterification, and hydroxypropylation, which alter the structure of the starch to reduce its in vitro digestibility, increase its water‐retention capacity, and lower its glycemic index (Nurmilah and Subroto 2021; Aparicio‐Saguilán et al. 2024). RS5 is formed by the interaction between amylose and lipid compounds during gelatinization (temperature > 80°C, lipid concentration 5%–7.5%, or use of moderate sonification), leading to the formation of a helical structure (V‐amylose), a complex that is resistant to enzymes (Yulianti 2022; Chen et al. 2024). Drying methods, particularly convection, can also increase the RS content of plantains by partially modifying the granular structure (Ávila‐Martín et al. 2025). Table 4 synthesizes these physicochemical indices and integrates them with their impact on RS formation and industrial utility.

**FIGURE 2:**
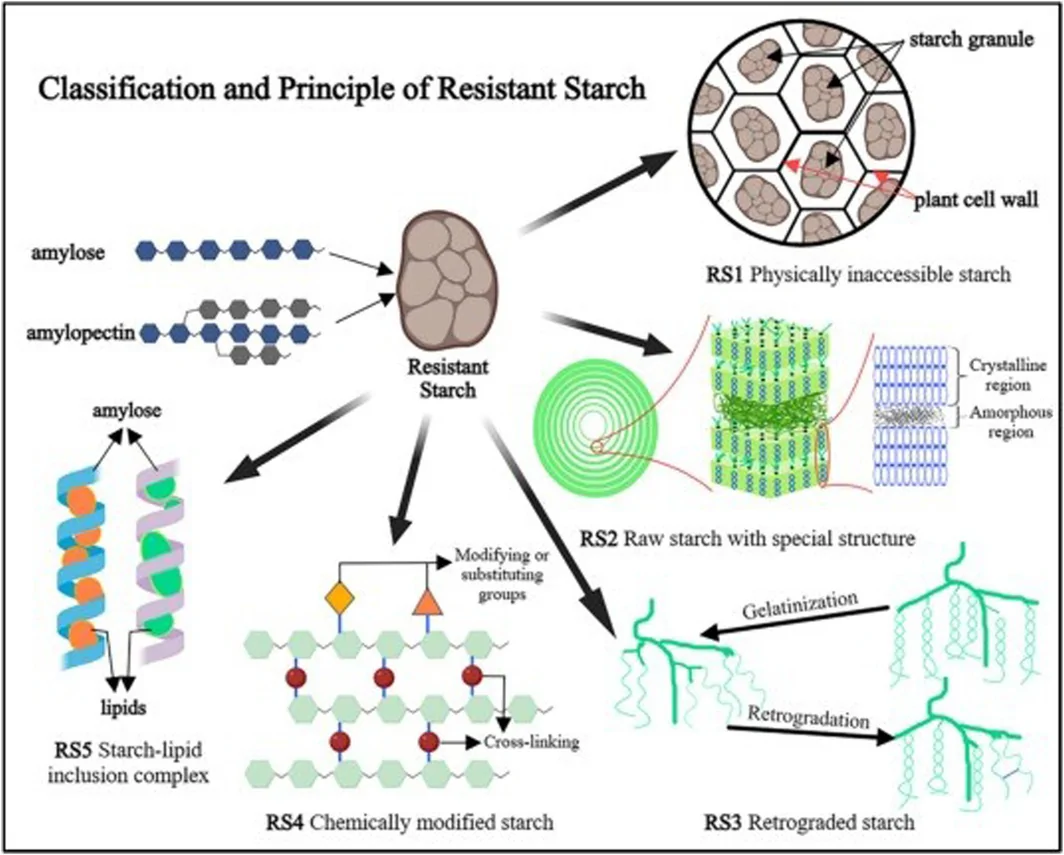
A diagram depicts different types of resistant starch fractions (Zang et al. 2024).

**TABLE 4:** Integrative analysis of physicochemical properties, RS fractions, and techno‐functional applications of PTR starches.

Starch source	Granule morphology & size	AM content (%)	Gelatinization (*T*_0_ − *T*_c_)/Δ*H*_gel_ (J/g)	Dominant RS type (Figure 2) & mechanism	Industrial & functional applications
Cassava ( *M. esculenta* )	Round/spherical (5–35 μm)	18.6–23.6	59.6°C–87.2°C/4.8	Low RS2/RS3: Rapidly digested due to branched amylopectin	Cohesive binders, thickeners, stabilizers; ideal for clear, elastic gels
Plantain (*Musa* spp.)	Irregular/polygonal (3.7–41 μm)	24.2–40.0	68.1°C–77.2°C/8.6–15.0	High RS2/RS3: Rigid gels; irregular shape impedes amylase access, high amylose promotes extensive recrystallization	Slow‐disintegration tablets; controlled‐release drug matrices
Potato ( *S. tuberosum* )	Spherical/oval (5–100 μm)	20.0–30.0	58.0°C–75.0°C/6.5	RS2/RS3: B‐type crystals; significant retrogradation upon cooling	Low‐glycemic foods; firm pharmaceutical capsules; dental adhesives
Sweet potato ( *I. batatas* )	Varied/polygonal (2–46 μm)	18.0–28.0	64.6°C–84.6°C/12.9	Modulated RS3: Retrogradation potential high via hydrothermal cycles	Functional baked goods; expansion agents in snacks; texturizers
Taro ( *C. esculenta* )	Round‐polygonal (0.5–5.0 μm)	14.0–19.4	81.0°C–85.0°C/6.8	RS1: Small, dense granules resist structural breakdown	Hypoallergenic infant formulas; aerosol glidants; sausage fillers
Yam ( *D. rotundata* /*alata*)	Oval/truncated (18–50 μm)	21.6–38.0	68.0°C–92.9°C/8.6–19.0	High RS2/RS3: High amylose promotes extensive recrystallization	Sustained‐release excipients; moisture‐stable thickeners for sauces

Abbreviations: Δ*H*_gel_, enthalpy of gelatinization; AM, amylose; *T*_0_ − *T*_c_, onset to conclusion gelatinization temperature.

#### 5.1.1 Influence of Amylose and Granule Morphology on Digestibility

The amylose/amylopectin ratio critically modulates starch digestibility and thermal stability. The digestibility of starch from roots and tubers (such as potatoes, cassava, and yams) is primarily governed by a combination of high amylose content and large, smooth granule morphology, which restricts enzymatic access compared to cereal starches. High‐amylose root starches and unripe plantain tend to have higher resistant starch content, resulting in a lower glycemic index and slower digestion (Van Hung et al. 2017; Varghese et al. 2023). Cassava starch, with its relatively low amylose and high amylopectin content, produces highly viscous gels suitable for use as cohesive binders in the pharmaceutical industry (Luo et al. 2022). In contrast, the high amylose content of yams (up to 38%) and green plantains favors the formation of RS2 and RS3, yielding firm, poorly digestible gels that act as effective matrices for sustained‐release drug delivery (Otegbayo et al. 2010).

Granule morphology further dictates the surface‐area‐to‐volume ratio available for enzymatic attack. The exceptionally small granules of taro (0.5–5 μm) provide a high surface area for rapid digestibility when the crystalline structure is disrupted, yet their dense polyhedral packing contributes to structural durability (RS1) in certain formulations (Zeng et al. 2014). In contrast, the larger, ovoid granules of yams and potatoes (10–50 μm) offer different hydration kinetics and enzymatic accessibility, influencing the rate of glucose release in the digestive tract.

#### 5.1.2 Morphological Influence on Hydration and Viscosity

The diversity in granular shapes, ranging from the polyhedral structures of taro to the spherical and elongated forms of cassava and yams, profoundly affects the surface‐area‐to‐volume ratio. Smaller granules, such as those found in taro (5 μm), provide an expansive surface area that facilitates rapid water absorption and enzymatic attack. This translates into high swelling power at lower temperatures compared to larger, more compact granules.

Conversely, the medium‐to‐large granules (> 10 μm) typical of certain yam species and plantains exhibit higher structural integrity. During processing, these larger granules tend to produce higher peak viscosities but are more susceptible to mechanical shear, leading to “viscosity breakdown” if not stabilized. The shape further influences the packing density of the starch matrix; polyhedral granules often result in more rigid, opaque gels, whereas spherical granules (like those in cassava) contribute to the characteristic clarity and smooth texture of the resulting paste (Pascoli Cereda 2023; Houngbo et al. 2024; Heider et al. 2025).

#### 5.1.3 Thermophysical Transitions: Gelatinization and Retrogradation

Gelatinization is a critical thermal transition (*T*_0_ to *T*_c_) influencing manufacturing robustness. Taro requires high temperatures (70°C–90°C) to reduce pathogens during pasteurization, a prerequisite for sterile pharmaceutical formulations. Retrogradation, the recrystallization of amylose and amylopectin, is a mechanism that occurs when cooling after the gelatinization phase. This process facilitates the formation of RS3 fractions, resulting in rigid gels that are ideal for sustained‐release drug delivery and low‐glycemic food products (Aboubakar et al. 2008; Otegbayo et al. 2010). Yams and plantains undergo intensive retrogradation, which is beneficial for controlled‐release matrices but may lead to syneresis in fresh food applications. Conversely, cassava's branched amylopectin structure decelerates retrogradation, ensuring the shelf‐life stability of semi‐solid oral formulations (Sánchez et al. 2010).

#### 5.1.4 Size‐Dependent Thermal Transitions: Gelatinization and Enthalpy

Size distribution is a primary determinant of the gelatinization temperature range. Small granules generally require higher thermal energy to disrupt their more uniform crystalline regions. For instance, taro starch requires a higher onset temperature (*T*_0_) for gelatinization than cassava. This correlation between granule size and thermal stability is critical for food safety, as it dictates the heat‐moisture treatments required to achieve a microbiologically stable and fully functionalized product.

The enthalpy of gelatinization (Δ*H*_gel_), reflecting the loss of molecular order within the granule, varies significantly across PTR species. High‐amylose starches (like plantain) often show higher resistance to swelling, which correlates with their role as functional fibers and resistant starches. This structural resistance is a double‐edged sword: while it provides health benefits via a lower glycemic index, it requires optimized industrial processing, such as pre‐gelatinization, to ensure consistency in formulations where high solubility is desired (Assemand et al. 2012).

### 5.2 Techno‐Functional Performance of Tropical Crops

Beyond their molecular architecture, PTRs exhibit macroscopic functional properties, water/oil absorption, swelling power, and rheological behavior that determine their culinary and industrial utility.

- *Absorption capacities*: Cassava starch exhibits exceptional water (0.77 g/g) and oil absorption (0.99 g/g) due to the abundance of hydrophilic and hydrophobic groups in its amylopectin chains. High oil absorption is a critical quality attribute for emulsification and reducing greasiness in fried products (Odi and Sinija 2021).
- *Swelling power and solubility*: Swelling power indicates the degree of hydrogen bonding between water and starch polymers. Cassava reaches maximum swelling (~40%) at 90°C, facilitating its use as an expansion agent in snacks. Solubility increases with thermal disruption of crystalline regions, aiding in the formulation of instant thickening agents.
- *Rheological and surface properties*: The storage modulus (G′) of starch gels follows the order *D. bulbifera* > *D. dumetorum* > *D. alata* , reflecting interspecific differences in gel firmness. Furthermore, the foaming capacity of cassava (7.5%) and the natural emulsifying properties of yam saponins enable the stabilization of oil‐in‐water nanoemulsions, offering plant‐based alternatives to synthetic surfactants (Schreiner et al. 2022).
- *Chemical modifications*: Functional properties can be further optimized via acetylation (increasing swelling) or acid hydrolysis (increasing powder compressibility and tablet hardness). Humidity‐heat treatments are employed to tailor viscosity and shear stability for high‐mechanical‐stress industrial processes.

These functional attributes position tropical starches as sustainable, gluten‐free alternatives to maize and wheat starches. By leveraging the specific physicochemical profiles outlined in Table 4, industry stakeholders can select the optimal PTR matrix for applications ranging from hypoallergenic infant formulas to advanced pharmaceutical drug delivery systems.

Ultimately, the transition from raw agricultural commodity to high‐value functional ingredient depends on the precise calibration of these structural parameters. Understanding these granular dynamics allows for the “rational design” of food products that meet both the sensory expectations of consumers and the rigorous stability requirements of modern food chains.

These intricate molecular and rheological attributes do not merely dictate fundamental pasting behaviors; they fundamentally establish the technological boundary conditions for food processing. The susceptibility of PTR starches to thermal shear, gelatinization enthalpy, and retrogradation kinetics determines the degree of cell disruption and starch solubilization required during industrial transformation. Consequently, translating these raw physicochemical dynamics into shelf‐stable, organoleptically accepted, and nutritionally enhanced foods requires precise thermal, mechanical, or biotechnical interventions, forming the continuum between ancestral processing techniques and contemporary agro‐industrial innovations examined in the following section.

## 6 Processing Innovations and Post‐Harvest Technology

The transformation of PTRs from highly perishable raw commodities into stable, palatable, and safe food products relies on a continuum of traditional and innovative technologies. Post‐harvest management is critical not only for extending shelf life but also for optimizing the interconnected nexus of functional quality, toxicological safety, and micronutrient retention mandated by contemporary food standards.

### 6.1 Traditional Processing and Preservation Methods

Traditional methods remain the bedrock of PTR utilization in tropical regions. These processes utilize thermal, physical, and biological agents to modify the starch matrix and eliminate endogenous antinutrients.

#### 6.1.1 Hydrothermal Treatments: Boiling and Steaming

Boiling remains the most ubiquitous processing technique, serving as a primary detoxification step, particularly for bitter cassava, where it facilitates the leaching of lethal cyanogenic glycosides into the aqueous phase (Perla et al. 2012). Although boiling effectively initiates starch gelatinization to improve digestibility, it is associated with significant micronutrient leaching, where water‐soluble vitamins (C and B‐complex) and minerals like potassium migrate into the discarded cooking water (Dong et al. 2024).

In contrast, steaming emerges as a superior nutritional alternative. By utilizing moist heat without direct water immersion, steaming preserves the mineral matrix and volatile flavor profiles of yams, sweet potatoes, and plantains more effectively than boiling (Buratti et al. 2020; Mundéné‐Timothée, Hitlamani, et al. 2025). From a structural standpoint, both methods soften the cellulose‐hemicellulose network, preparing the tubers for mechanical reduction.

#### 6.1.2 Mechanical Functionalization: Pounding and Grinding

Pounding is a high‐shear mechanical process that converts gelatinized starch into elastic, cohesive doughs (e.g., *fufu* or pounded yams). This step is crucial for modifying the rheological properties of the starch, as physical force breaks down cellular aggregates, producing a smooth, “long” texture favored in tropical diets (Elisabeth et al. 2022). This mechanical energy disrupts the semi‐crystalline starch structure, enhancing the techno‐functional elasticity of the food (Akinoso and Abiodun 2016). For flour production, grinding pre‐dried chips into fine powders increases the surface area, which improves the hydration kinetics and functional performance of the resulting flours in composite food systems in subsequent baking or extrusion applications (Achi 1991).

#### 6.1.3 Dry Heat and Lipid‐Based Transitions: Roasting, Baking, and Frying

Roasting and baking utilize dry heat to induce the Maillard reaction and caramelization, creating complex aromatic profiles and a characteristic organoleptically desirable crust characterized by pyrazine and caramel aroma volatiles. Although these methods enhance sensory appeal, they also lead to the denaturation of proteins and partial dehydration, resulting in a firmer, drier texture (Tsai et al. 2021), and necessitate caution regarding chemical safety; improper roasting can lead to the formation of polycyclic aromatic hydrocarbons (PAHs) (Abiona et al. 2021).

Frying operates at higher temperatures (160°C–190°C), acting as a dehydration‐impregnation process where water is replaced by lipids, creating a crisp barrier. The immersion in hot oil creates a lipid barrier that reduces water activity (*a*_w_), effectively serving as a partial preservation technique (Reyniers et al. 2020; Chang et al. 2020). However, the high‐temperature reaction between reducing sugars and amino acids (asparagine) in starchy tubers poses a risk of acrylamide formation. This risk is mitigated in industrial and traditional settings through pre‐frying, soaking, or blanching (60°C–70°C), which leaches out the precursor sugars (Kansci et al. 2016; Ngobese and Workneh 2018).

#### 6.1.4 Biochemical and Environmental Preservation

Fermentation is perhaps the most sophisticated traditional technology, acting as a spontaneous microbial biocatalytic process for detoxification, flavor development, and nutritional enrichment. By utilizing indigenous lactic acid bacteria (LAB) and yeasts that break down carbohydrates in cassava processing, such as producing gari or lafun, fermentation lowers the pH, facilitating the enzymatic breakdown of linamarin (a cyanogenic glycoside) and inhibiting spoilage organisms (Iwuoha et al. 2014; Halake and Chinthapalli 2020). Beyond safety, fermentation enriches the matrix with B‐group vitamins and improves mineral bioavailability by degrading phytates (Halake and Chinthapalli 2020).

Sun‐drying remains the most cost‐effective method for long‐term preservation by reducing water activity (*a*_w_) to levels that inhibit microbial proliferation. However, direct exposure to UV radiation can degrade light‐sensitive compounds like β‐carotene in orange‐fleshed sweet potatoes, suggesting a need for solar‐drying innovations that protect nutritional integrity (Menya et al. 2024).

#### 6.1.5 Post‐Harvest Management: Underground Storage

Ingenious traditional methods like underground storage (pits or clamps) exploit the soil's thermal inertia to maintain a stable microclimate. This delays physiological deterioration and sprouting in yams and potatoes by regulating the ambient humidity and temperature (Stephen et al. 2022). Although effective for tubers, this method is unsuitable for climacteric fruits like plantains, which require more sophisticated atmospheric control to manage ethylene‐induced ripening.

#### 6.1.6 Synthesis of Processing Impacts

Table 5 summarizes the trade‐offs between traditional processing methods and their impact on food quality and safety. The transition from these traditional labor‐intensive methods toward modern processing innovations, such as flash drying, extrusion, and high‐pressure processing, is essential to scale up the utilization of PTRs in the global food industry while maintaining their functional and nutritional “gold standard.”

**TABLE 5:** Impact of traditional processing methods on food quality and safety.

Method	Primary goal	Nutritional impact	Safety benefit
Boiling	Softening/cooking	Leaching of vitamin C & B	Cyanide reduction
Steaming	Texture/cooking	High nutrient retention	Minimal
Fermentation	Detoxification/flavor	B‐vitamin synthesis	Pathogen inhibition
Frying	Palatability/snack	Lipid uptake; acrylamide risk	Moisture reduction
Sun‐drying	Shelf‐life	Potential UV‐degradation	Microbial stability

### 6.2 Modern Technological Innovations in Root, Tuber, and Plantain Processing

The transition from traditional post‐harvest management to modern agro‐industrial valorization of PTRs represents a significant shift toward enhancing food quality and safety. Although traditional methods like boiling and sun‐drying are essential for basic detoxification and stabilization, modern techniques offer high‐precision control over the molecular and nutritional integrity of these crops. This section explores how advanced technologies, ranging from thermal extrusion to nanotechnology, address the limitations of ancestral practices by improving digestibility, preserving heat‐sensitive bioactives, and creating high‐value functional ingredients.

#### 6.2.1 Thermomechanical Transformation: Extrusion Technology

Extrusion stands as a versatile pillar of modern PTR processing, facilitating the transformation of flours into diverse textured products such as puffed snacks, breakfast cereals, and even meat analogues (Sule et al. 2024). This continuous process involves subjecting starch‐rich pastes to high pressure, mechanical shearing, and elevated temperatures within a machine barrel (Yadav et al. 2025). As the material is forced through a die, the instantaneous drop in pressure generates characteristic porous and expanded structures.

The complexity of this technology is detailed in Figure 3, which classifies extruders based on screw type (single vs. twin), operational mode (hot vs. cold), and mechanism (piston vs. roller) (Yadav et al. 2025). Twin‐screw extruders, in particular, offer superior control over mixing and shearing, making them ideal for developing gluten‐free pasta from cassava or nutrient‐dense granules from potatoes (Gborie et al. 2022; Yadav et al. 2025). Beyond texturization, extrusion improves starch digestibility and extends shelf life through controlled dehydration, providing a robust solution for the agro‐industrial valorization of tropical staples (Sule et al. 2024; Yadav et al. 2025).

**FIGURE 3:**
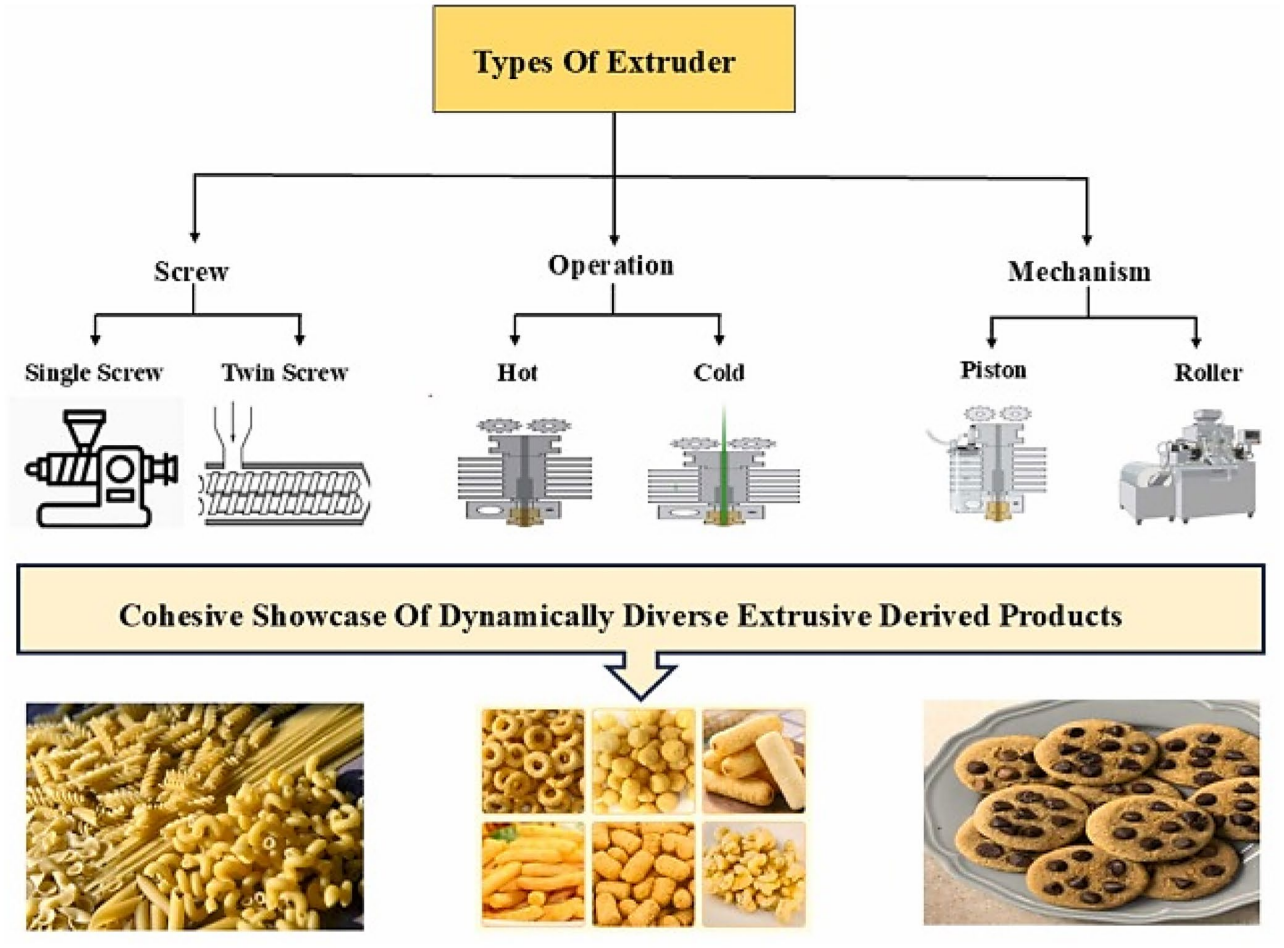
Classification of extruder technologies: Screw type, operational mode, and mechanism, with corresponding extruded products (Yadav et al. 2025).

#### 6.2.2 Advanced Dehydration and Microbial Stabilization

Where traditional sun‐drying is limited by weather dependence and potential UV degradation of pigments like β‐carotene, modern dehydration techniques offer precision. Freeze‐drying (lyophilization) utilizes the principle of sublimation, transforming ice directly into gas under a vacuum, to preserve the original color, aroma, and delicate cellular structure of PTRs (Bhatta et al. 2020; Gat and Gawande 2024). This “cold dehydration” is essential for high‐value applications, such as space food derived from sweet potatoes or gourmet taro ingredients, where nutritional stability is paramount (Bhatta et al. 2020; Prosapio and Lopez‐Quiroga 2020).

Complementing this is high‐pressure processing (HPP), a non‐thermal pasteurization technique (Javed et al. 2023). By subjecting pre‐packaged PTR products to hydrostatic pressures between 400 and 600 MPa, HPP irreversibly disrupts microbial cell membranes without the destructive effects of high heat (Iqbal et al. 2022). This preserves the “fresh‐like” quality and heat‐sensitive vitamins in products such as mashed plantains and sweet potato‐based baby foods, offering a safer, high‐quality alternative for the refrigerated ready‐meal market (Wei et al. 2022; Gokul Nath et al. 2023).

#### 6.2.3 Biotechnological Interventions: Enzymatic and Probiotic Modification

Modern biotechnology enhances the functional versatility of PTR starches through targeted enzymatic modification (Wang et al. 2020). Unlike traditional pounding, which relies on physical force, enzymes such as maltogenic amylases, branching enzymes, and β‐amylases, as shown in Figure 4a, hydrolyze specific glycosidic bonds to tailor starch properties (Amaraweera et al. 2021; Punia Bangar et al. 2022). Figure 4b illustrates these mechanisms: α‐amylases cleave internal α‐1,4‐glucosidic bonds, whereas pullulanases target α‐1,6‐glycosidic branches in amylopectin to produce linear amylose chains. Such modifications improve thickening stability and freeze–thaw resistance in potato‐based frozen sauces and cassava‐derived textiles (Choton et al. 2024; Zang et al. 2024).

**FIGURE 4:**
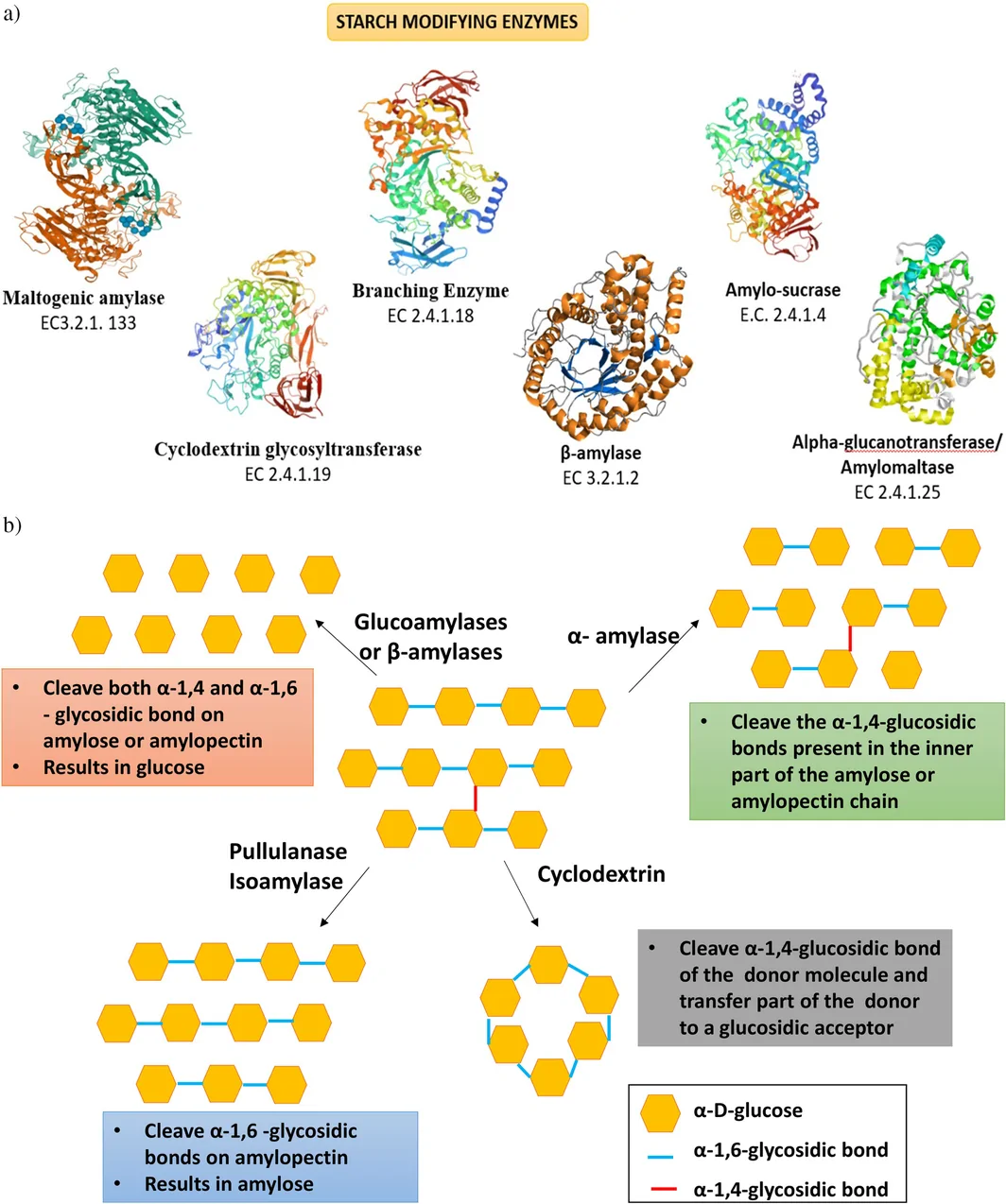
Various starch‐modifying enzymes (Amaraweera et al. 2021; Punia Bangar et al. 2022). (a) Main enzymes used for enzyme modifications. (b) Mechanism of action of enzymes on starch.

Furthermore, probiotic fermentation merges ancestral knowledge with modern microbiology (El‐Sohaimy and Hussain 2023). By inoculating tuber purees with selected *Lactobacillus* spp. under sterile conditions, processors can transform traditional staples like *gari* into functional foods. This process not only synthesizes essential B‐vitamins and bioactive peptides but also significantly reduces antinutrients, thereby enhancing mineral bioavailability and promoting intestinal health (Dahiya and Nigam 2022; El‐Sohaimy and Hussain 2023).

#### 6.2.4 Nanotechnology and Pulse‐Based Innovations

At the cutting edge of PTR valorization lies encapsulation nanotechnology, which addresses the fragility of bioactive compounds (Ansari 2023). Sensitive molecules like provitamin A from sweet potatoes or anthocyanins from purple yams are confined within nanovectors. Figure 5 highlights the diverse classes of these carriers, including lipid‐based liposomes, polymeric nanospheres, and nanocrystals, each offering specific advantages in payload flexibility or release control (Altemimi et al. 2024). As summarized in Figure 6, nanotechnology impacts the entire food cycle, improving nutritive quality through nanoencapsulation, enhancing food safety via intelligent antimicrobial packaging, and reducing spoilage through nanosensors (Abinash et al. 2021; Ansari 2023).

**FIGURE 5:**
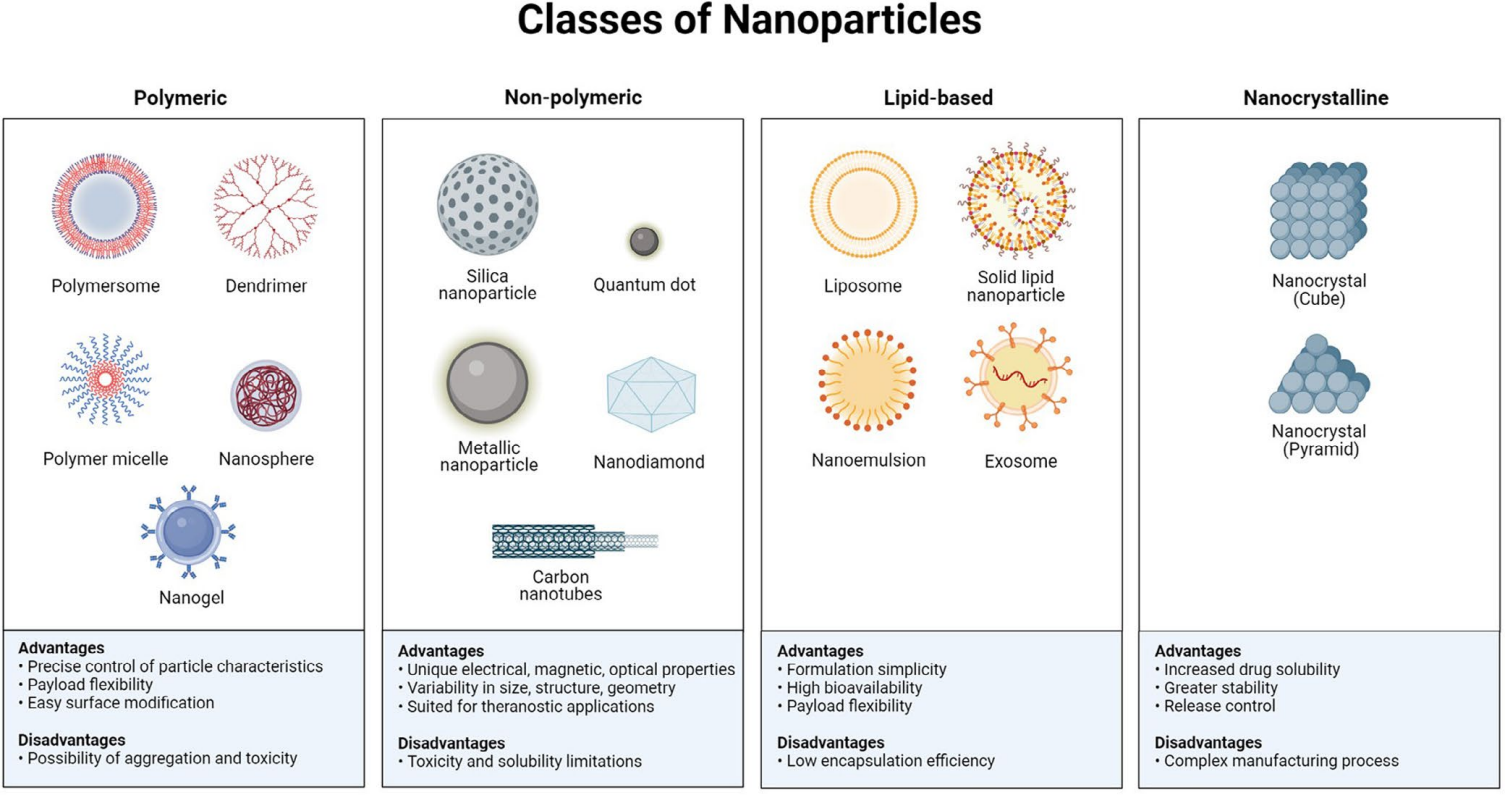
Main classes of nanoparticles (NPs), including polymeric, non‐polymeric, lipid‐based, and nanocrystalline materials for encapsulating nutrients and bioactive compounds from foods (Altemimi et al. 2024).

**FIGURE 6:**
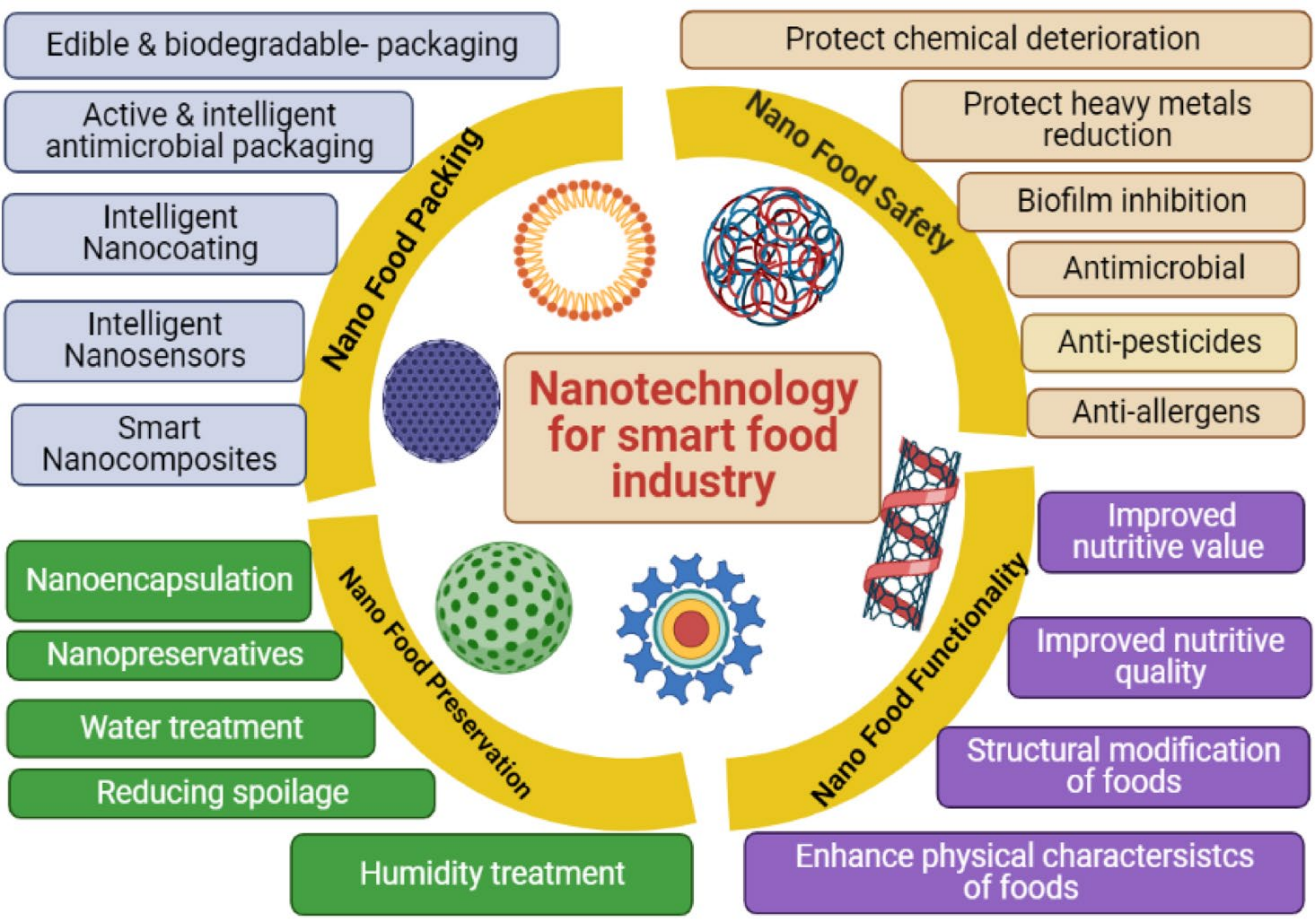
Application of nanotechnology in different areas of the food industry (Ansari 2023).

Finally, pulsed electric field (PEF) processing introduces an eco‐efficient dimension to PTR technology (Kumar et al. 2021). By applying brief electrical pulses (1–10 kV/cm), PEF induces electroporation in tuber cell membranes. This non‐thermal permeabilization facilitates the targeted extraction of starches and sugars while reducing energy consumption during subsequent drying by up to 40% (Kumar et al. 2021; Pateiro et al. 2021). PEF is particularly promising for improving the texture of potato fries and accelerating the detoxification of cassava.

#### 6.2.5 Integrated Processing Continuum: Synergizing Traditional Practices, Industrial Processing, and Emerging Technologies

From ancestral know‐how to cutting‐edge biotechnology, the processing of roots, tubers, and plantains is based on strategic complementarity: traditional methods (fermentation, solar drying, and pounding) form coherent systems optimized for detoxification, preservation, and the creation of iconic textures such as *fufu*, *foutou*, *ntuba ekōn*, *mitumba*, and *achu*, whereas relying on local, energy‐efficient resources. Sequential processing systematically incorporates several techniques, as illustrated by the processing chains for:

1. $\text{Cassava}:\text{Peel}\to \text{Grate}\to \text{Ferment}\;\left(\text{optional}\right)\to \text{Press}\to \text{Roast}\;\left(\text{gari}\right)\;\text{or Boil}\to \text{Pound}\to \mathrm{Eat}\;\left(\text{fufu}\right)\;\text{or Boil}\to \text{Ferment}\to \mathrm{Sun}-\mathrm{dry}\to \text{Pound}\to \text{Flour}$.
2. $\text{Yams}:\text{Harvest}\to \text{Store}\;\left(\text{underground}\right)\to \text{Peel}\to \text{Boil}\to \text{Pound}\to \mathrm{Eat}\;\left(\text{fufu}/\text{pounded}\;\mathrm{yam}\right)$.
3. $\text{Plantains}:\text{Peel}\to \text{Steam}/\text{Boil}\to \text{Pound}\to \mathrm{Eat}\;\left(\text{fufu}/\text{ntuba ek}\overset{®}{\text{o}}\text{n}\right)\;\text{or Slice}\to \mathrm{Sun}-\mathrm{dry}\to \text{Grind}\to \text{Flour}$.

The processing of roots, tubers, and plantains has evolved into a sophisticated integrated multi‐technology continuum. Traditional methods ensure detoxification and the preservation of cultural culinary identities. Modern techniques like HPP and extrusion enhance safety and enable industrial‐scale production. Emerging approaches like nanotechnology and PEF offer precision and sustainability. Although challenges such as high equipment costs and the need for standardized regulatory frameworks persist, this technological continuum is essential for meeting global imperatives of food safety, sustainability, and nutritional optimization (Ansari 2023; Altemimi et al. 2024). It is now imperative to systematically analyze how these multi‐scale interventions collectively modulate nutrient bioavailability and long‐term consumer health.

This processing continuum underscores that emerging non‐thermal and thermomechanical innovations do not render ancestral practices obsolete; rather, they serve complementary roles. Although traditional unit operations (soaking, spontaneous lactic fermentation, and solar drying) provide accessible, low‐cost baseline detoxification, modern interventions (HPP, precision extrusion, and encapsulation) offer the standardized molecular control necessary to preserve labile micronutrients and satisfy rigorous commercial standards. The ultimate success of these combined processing paradigms is manifested in the diverse portfolio of traditional, biofortified, and value‐added food products currently entering domestic and international markets, whose safety and functional profiles are evaluated below.

## 7 Applications in Food: Nutritional Security, Cultural Identity, and Agro‐Industrial Valorization

The processing and consumption of major tropical PTRs represent a dynamic intersection between ancestral heritage and modern food science. Although the potato remains the global benchmark for tuber consumption in temperate zones, crops such as cassava, sweet potato, taro, yam, and plantain serve as indispensable pillars of nutritional security for millions in tropical and subtropical regions (Varin 2018; CIRAD 2023). The transition from traditional stabilization to high‐value food applications is not merely a culinary evolution but a technological necessity to ensure food safety and market competitiveness.

### 7.1 Detoxification and Safety: The Technological Prerequisite

For several PTR species, successful food application and commercialization are strictly governed by the biochemical necessity to neutralize hazardous endogenous antinutritional factors (ANFs) and toxic secondary metabolites. Raw roots, tubers, and plantains contain variable concentrations of toxic substances, notably cyanogenic glycosides in cassava, steroidal glycoalkaloids in potato, and acrid calcium oxalate raphides in taro and yam that pose acute and chronic health hazards if not effectively managed (Codex Alimentarius 1989a, 1989b; EFSA CONTAM Panel 2019, 2020; Borku et al. 2025). As detailed in Table 6, transforming these potentially toxic crops into safe, shelf‐stable, and culturally accepted staples requires precise unit operations calibrated to the degradation kinetics of each target hazard.

**TABLE 6:** Critical inventory of traditional dishes and value‐added food products derived from PTRs.

Dish/product	Preparation method	Nutritional/functional value	Advantages	Limitations/drawbacks	References
Critical inventory of cassava‐based products					
Agbeli Kaklo	Grated cassava, 3–5 days of fermentation, mixed with spices, shaped, and deep‐fried	Carbohydrates, fiber, lipids (depending on oil), high energy	Traditional snack, good shelf life, local economic value	High fat content, artisanal/non‐standardized process	Nsa Oduro (2016)
Ayigbe biscuit	Mixed cassava flour (dry starch), sugar, nutmeg, milk, egg; molded and baked	Complex carbohydrates, long shelf life, low moisture	Appreciated sweet flavor, extended shelf‐life, gluten‐free	Low protein content, dense, high in calories	
Abacha (African salad)	Grated cassava, chips; palm oil/spices, vegetables (ugba, onions, various leaves), served cold or warm	Fiber, carbohydrates, micronutrients (per veg/fish added)	Versatile garnish, cultural appeal, micronutrient source	Non‐standardized, microbial contamination risk, variable nutrition	
Fried cassava chips	Fresh roots sliced, blanched or soaked, deep‐fried in oil	Carbohydrates, fiber, energy, and quality vary by method	Popular snack, good acceptance, alternative to potato chips	Micronutrient loss, high fat content, and product uniformity issues	
Peujeum (fermented cassava)	Peeled cassava, steamed, cooled, fermented with ragi starter, wrapped in leaves, 2‐day fermentation	Carbohydrates, moderate acidity, low alcohol, natural probiotics	Unique flavor, product diversity, improved digestibility	Limited starter access, not universally liked, food safety concern	
Pappad	Gelatinized flour, spread and sun‐dried, stored, and deep‐fried when consumed	Carbohydrates, crunchy texture, high digestibility	Ready‐to‐eat snack, long shelf‐life, easy distribution	Requires frying to eat, fat content	
Akara‐akpu (cassava balls)	Cassava flour/mash, spices, onion, salt, shaped and fried	Carbohydrates, protein enrichment if soy/melon added	Quick snack, possible enrichment, good shelf‐life	Low protein unless enriched, high in fat	
Gari (roasted grits)	Grating, fermentation, pressing, sieving, and roasting on a hot plate	Complex carbohydrates (~80%+), fiber, low protein/fat	Very long shelf‐life, versatile as a dish or snack	High glycemic index, lacks key micronutrients unless complemented	
Eba/gari fufu	Gari was added to hot water and stirred into a firm dough	Carbohydrates, energy, easily digested	Easy preparation, appealing texture, fits varied diets	Low nutrient density, needs supplementary foods	
Gari soakings	Gari soaked in cold water, sugar/milk/nuts sometimes added	Carbohydrates, fiber, lipids (per add‐ins)	Practical, socially accepted, adaptable to means	Can be low in protein and micronutrients	
Kpokpo gari	Similar to gari but unsieved before roasting, forms amorphous cakes	Carbohydrates, fiber, energy, and protein, depending on the garnish	Textural variation, long conservation, varied usage	Less refined, variable texture, possible hygiene issues	
Attieke	Grated cassava, short fermentation, sifting, steamed	Easily digested carbs, fiber, and enhanced by fermentation	Consumed hot or cold, couscous‐like texture, and cultural importance	Requires fermentation skill; steaming tech is not universal	
Kokonte/lafun	Roots dried (kokonte), or fermented/dried then milled (lafun), are both cooked into a paste	Carbohydrates, fiber, protein per variety, energy	Easy prep, suits diverse main dishes	Sometimes stigmatized as “poor man's food,” fermentation is not always controlled	
Placali	Fermented cassava flour is added to boiling water and stirred to a creamy consistency	Carbohydrates, improved digestibility, soft texture	Sensory variety, high acceptance in Côte d'Ivoire	Variable textures and value, needs quality flour	
Chickwangue	Peeled roots, fermented, dehydrated, wrapped in leaves, steamed	Carbohydrates, fiber, and protein, if garnished	Good shelf‐life, suitable for rural areas	Slow process, risk of contamination, and leaf availability	
Cassava dough	Grated roots, long fermentation, packed, used in breads/porridges/banku/akple	Carbohydrates, fiber, and acidity vary; value depends on use	Multipurpose, forms the base of many traditional meals	Variable fermentation quality, short shelf‐life	
Boiled cassava root	Boiled roots served with sauces/garnish, or incorporated in fufu/other meals	Carbohydrates, fiber, very low protein/fat	Simple preparation, accessible base food, aids digestion	Low nutrition except for sides/sauces	
Cassava leaves soup	Chopped leaves cooked/stewed with meat/peanut/fish	High protein (29%–32%), micronutrients, vitamin C	Strong protein source, deep African culinary tradition	High cyanogenic potential, needs long cooking	
Cassareep	Bitter cassava juice, boiled down, spiced, thickened into syrup	Fewer cyanogens, antioxidants, and antiseptic properties	Long preservation, culinary/medicinal uses, sauce base	Very lengthy process, needs specific bitter varieties	
Chicha/mingao	Fermented cassava, brewed as beer, sometimes fruit/spice flavored	Fermented carbohydrates, alcohol, fiber, and possible probiotic content	Industrial value‐added, extra nutrition	Quality control challenge, potential alcohol/microbial risks	
Composite breads & pastas	Cassava flour (up to 30%) blended with wheat, margarine/egg, baked	Gluten‐free, improved digestibility, varied textures	Alternative for gluten‐sensitive, local sourcing	Structure/volume issues, margarine/egg cost, needs recipe tuning	
Baby foods	Fermented/non‐fermented cassava, soy, fruits, cooked/extruded	Easily digested, slow energy, protein/micronutrient enrichment	Allergy‐safe for infants, dietary variety	Lack of standardization, fermentation risk, variable texture	
Critical inventory of plantain‐based products					
Fried plantain (alloco)	Ripe plantain slices are deep‐fried until golden, salted, and drained	High in complex carbohydrates, moderate fiber	Crispy snack, quick side dish	High fat from frying	Newilah et al. (2005) and Mundéné‐Timothée et al. (2024)
Plantain purée	Ripe plantain is boiled until soft, mashed with butter or oil, and seasoned	Long‐lasting energy, rich in fiber	Alternative to potato purée, digestible	Lower protein, less firm texture	
Baked plantain	Halved ripe plantain brushed with olive oil and spices, baked 25 min at 400°F	Lower in fat than the fried version, rich in fiber	Lighter dish, preserves nutrients	Longer cooking time	
Plantain gratin	Plantain rounds with light cream and cheese, baked 20 min at 355°F	Source of calcium and cheese protein	Comfort food, filling	Higher calories require an oven	
Tostones (green plantain)	Green plantain sliced, briefly fried, smashed, then fried again until crisp	Complex carbs, low sugar	Crunchy snack, unique texture	Double frying, higher fat	
Sautéed plantain with garlic & parsley	Ripe plantain slices sautéed in oil, finished with garlic and parsley	Fiber, natural aroma	Light, tasty side	Must be eaten fresh	
Plantain fritters	Very ripe plantain mashed, mixed with flour, egg, sugar (option), and fried in small portions	Quick energy, sweet taste	Popular snack or dessert	High‐calorie and fat intake	
Plantain soup	Green plantain rounds cooked in broth with onion, garlic, blended	Nutritious, complex carbs	Light, gentle flavor, hydrating	Little protein	
Warm plantain salad	Steamed ripe plantain slices tossed with cherry tomato, onion, and lemon vinaigrette	Fiber, vitamins, antioxidants	Refreshing, sweet/savory dish	Needs prep	
Grilled plantain (BBQ)	Split ripe plantain grilled, brushed with oil/honey, optionally	Fiber, complex carbohydrates	Caramelized, simple, tasty dish	Risk of burning	
Boiled plantain	Unripe/ripe plantain boiled (often with salt), eaten with beans, sauces, or meats	Main source of carbohydrates, moderate fiber	Most common household dish, pairs well	May lack protein alone	
Dried plantain/banana	Unripe or semi‐ripe plantain dried at 150°F for 48 h or sun‐dried for 2–3 days	Energy‐dense, longer shelf‐life	Export product, shelf‐stable	Requires equipment or sun, not a complete meal	
Plantain chips	Sliced unripe plantain fried in palm oil at 320°F, packed for local/international consumption	High in carbohydrates, some fiber	Popular snack, source of income (for women)	High‐fat, processed food, and possible additives	
Fried plantain (ripe)	Sliced ripe plantain fried in palm/vegetable oil at 320°F, served with omelet, veggies, and meats	High carbohydrates, sugar for energy	Celebratory meal, liked by children	High‐fat; not suitable daily for all diets	
Roasted plantain	Ripe/unripe plantain roasted over charcoal, served with plum, pear, fish, meats, veggies	High carbohydrates, fiber, and variable micronutrients	Economic street food, widely affordable	Risk of contamination; limited shelf‐life	
Traditional plantain purée	Overripe plantain boiled with garden egg, hot pepper, palm oil, and mashed	Easily digestible, fiber, vitamin A/C	Reduces waste, suitable for the elderly	Not high in protein	
Malaxé (mashed plantain with nuts or beans)	Unripe plantain sliced and boiled, mashed with peanut, soybean, or egussi paste, onion, and fish	Higher protein, fiber, micronutrient‐rich	Economic dish, uses plant protein, satiating	Preparation time may require many ingredients	
Bouillon “Douala‐midi”	Unripe plantain w/onion, leek, dried fish, crayfish, palm oil, and spices, boiled	Hearty, protein from fish, complex flavor	Quick prep, deeply flavored traditional food	Multiple ingredients, sodium content	
Mielemassesse (sautéed plantain)	Boiled unripe plantain chunks mixed with palm oil	Energy, vitamin A from oil	Ritual dish, prepared for celebrations	Needs fresh oil, prone to spoilage	
Kondre (plantain with meat)	Whole unripe plantain boiled ~3 h with meats, tomato, onion, spices	Complete meal: carbohydrates, protein, vit A/B	Special occasion dish, festive, deeply nourishing	Long preparation, expensive ingredients	
Ntuba ekōn (plantain dough)	Boiled unripe plantain pounded to a thick dough, sometimes mixed with sauces	High satiety, fiber, complex carbohydrates	Staple food, ceremonial; pairs with Ndô/pepper/groundnut	Labor‐intensive, plantain shortages, and storage	
Plantain paste with beans/green vegetables	Boiled unripe or ripe plantain, pounded and mixed with kidney beans or green vegetables	More protein, minerals, vitamins	Children's favorite, complete meal	Long preparation, multiple steps	
Ngomezeck	Unripe plantain sliced, mixed with egussi paste, dried fish, pepper, salt, wrapped in leaves & boiled	Satiating strengthens protein and fat intake	For the elderly, ceremonial, appealing for micronutrients	Many ingredients need a wrapping technique	
Plantain flour	Unripe (sometimes ripe) plantain sliced, blanched, dried < 150°F, ground to flour	Source of gluten‐free carbs, minerals, and fiber	Used in baking, fritters, cakes, and storable foods	Not a complete protein, needs complementary ingredients	
Critical inventory of sweet potato‐based products					
Sweet potato bread	Addition of sweet potato flour to the dough, 2 fermentations, baking at 200°C–220°C	Provides fiber, minerals, lower cost, and lower Glycemic Index than wheat	Reduces cost, unique taste, better texture	Too much sweet potato = dense texture, dosing is difficult	Mu, Sun, and Li (2016) and Mu, Li, and Sun (2016)
Sweet potato cookies	Cooked sweet potato mixed into dough, extruded, quick high‐temp baking	Fiber, natural sweet taste, less added sugar	Novel taste, fiber, low‐fat content	Too much sweet potato = dough hard to handle	
Purple sweet potato cake	Mix flour, eggs, purple sweet potato; whip, mold, and bake at 175°C–180°C	Antioxidants, anthocyanins, moderate Glycemic Index	Attractive color, health potential, marketable	Higher price, color/texture sensitive	
Instant nutritious sweet potato chips	Wash, peel, slice, pre‐gelatinize, roller dry, granulate	Rich in complex carbohydrates, vitamins, and easy to store	Healthy snacks, fast energy, and long preservation	Industrially complex process	
Puffed sweet potato snacks	Dried sweet potato (80%) + rice/millet, extruded puff	Crunchy, intermediate Glycemic Index, more fiber than corn	Not fried, low‐fat, modern method, nutritious	Puffing is tricky to master	
Airflow puffed sweet potato chips	Partial dehydration, pressure cooking, then rapid air blast, dried and nitrogen‐packed	Crunchy, low moisture, moderate energy	Efficient (time/energy saving), preservable	High equipment cost, technical mastery needed	
Aromatic, crispy sweet potato chips	Cook, short fermentation, drying, cutting, short bake, and fry	Moderate energy, natural aromas, light texture	Enhanced flavor, better shelf life	Standardizing texture/fry quality is challenging	
Low‐temp vacuum fried sweet potato chips	Blanched slices, dip, vacuum fry at 85°C, centrifuge de‐oil	Less fat than regular fries, preserves nutrients	Optimal taste, low fat, good crisp/texture	Expensive equipment, slow process	
Vacuum‐microwave dried sweet potato chips	Blanch, freeze, pre‐dry, vacuum microwave dry	High nutritional value, airy and light texture	Fast drying, crispy, good color	Needs specialized equipment	
Sun‐dried sweet potato slices	Boiled, peeled, sliced, sun‐dried, and stored for natural sugar	Rich in natural sugars, fiber, and micronutrients	Little processing, long shelf life	Weather‐dependent, contamination risk	
Sweet potato jelly	Cooked, blended, gelled (konjac/carrageenan), portioned	Energy, sweet taste, appealing texture	Novelty, no artificial flavors	Precise dosing needed for ideal texture	
Instant sweet potato noodles	Extracted starch, extruded, dried, ready after soaking	Moderate Glycemic Index, gluten‐free, fiber, digestible	Quick prep, convenient	Lacks flavor unless seasoned	
Frozen sweet potato products	Cut, steamed, quickly frozen	Preserved vitamins/minerals, ready to eat	Convenient, long shelf life	Requires a cold chain	
Sweet potato leaf health tea	Young leaves, low‐temp dried, blended	Polyphenols, antioxidants, fiber	Pleasant, healthy drink, new market	Spoils easily, strong taste	
Canned sweet potato shoots	Young shoots, blanched, anti‐browning, sterilized	High in fiber and micronutrients	New use for the plant, long storage	Requires industrial processing	
Sweet potato beer	Liquefied starch, fermented at low temp., filtered, and pasteurized	Natural ferment, lower glycemic index than regular beer	Unique taste, agro‐food diversification	Demanding process, small market	
Purple sweet potato juice	Enzyme extraction, clarification, and pasteurization	Antioxidants, low‐calorie, natural color	Health benefit, attractive color	Special taste, short shelf life	
Whole sweet potato flour	Peeled, dried, ground	High functional value, includes all but skin	High potential, gluten‐free	Limited knowledge, applications development	
Sweet potato protein	Extracted from starch wastewater by foam separation. Rich in essential amino acids (40.7%)	High‐quality plant protein. Excellent functional properties (solubility, emulsification)	Valorization of effluent reduces pollution. Increases added value.	Conventional techniques (biochemistry, membranes) are costly and complex	
Dietary fiber	Extracted from sweet potato residues (after starch extraction) by enzymatic hydrolysis (thermostable α‐amylase) followed by drying	Content > 20%. It can regulate nutrient absorption	Valorization of co‐products. Important for nutritional balance	The official AOAC enzymatic‐gravimetric method is lengthy and costly	
Sweet potato pectin	Extracted from dietary fiber by acid extraction, concentration, ethanol precipitation, and drying. Content of 20%–30% in residues	Physiological activities: fat metabolism, gut flora, and anti‐cancer	Alternative source to citrus and apples. Wide applications (food, pharma)	Conventional extraction (acid, 90°C) easily degrades pectin	
Anthocyanins (purple SP)	Extracted by ethanol‐ammonium sulfate aqueous two‐phase (ATPE) system. Heat and light stable	Natural pigments with strong antioxidant properties. Proven absorption in vivo	Abundant source and reasonable price. Superior stability to other pigments	Polymer ATPE systems (PEG/salt) are costly and difficult to handle on a large scale	
Polyphenols (leaves)	Extracted from leaves by ultrasound with 70% ethanol, purified on AB‐8 macroporous resin. Rich in chlorogenic acid	Antioxidant, antibacterial, anti‐inflammatory, and anti‐tumor activities	Valorization of leaves. Broad applications (food, cosmetics, pharma)	Conventional extraction methods are lengthy and costly	
Sweet potato leaf powder	Fresh leaves washed, dried, and finely ground. No additives	Rich in proteins, polyphenols, dietary fiber, and minerals	Easy‐to‐incorporate vegetable nutritional supplement in various foods	Processing and storage conditions of existing products lead to nutrient losses	
Critical inventory of taro‐based products					
Poi (fermented taro paste)	Boil corms, peel, mash, dilute, acidify with lemon; natural fermentation 2–3 weeks	Complex carbohydrates, fiber, rich in probiotics (lactic fermentation), vitamins, and minerals	Long shelf‐life (> 1 month), improved digestibility, hypoallergenic	Acidic flavor, microbial risk if pH is too high, mainly for traditional ceremonies	Njintang et al. (2016)
Sapal	Cooked taro, peeled, grated, mixed with coconut cream, fermented at room temperature	Energy, prebiotics, sweet flavor, positive lactic bacteria	Widely preferred taste, local value, social use	Risk of microbial contamination if poorly fermented	
Taro ice cream/ice bar	Cooked taro, pureed or flour, blended with fats, sugar, stabilizers, frozen	Carbohydrates, starch, vitamins, low allergenic potential, soft or firm texture (depending on type)	Industrial diversification, promising dairy substitute	High in sugar and fat, flavor may not appeal to all; texture depends on the process	
Taro yogurt (Taro Gurt)	Taro fermented with specific LAB strains isolated from poi	Probiotics, gut health support, rich in fiber and carbohydrates, B vitamins	Boosts digestive health, innovative texture, and active research for fortification	Difficult acid control, low bacterial survival at low pH, sometimes stiff texture	
Taro noodles	Partial substitution of wheat or rice flour with taro flour, extrusion, and cooking	Gluten reduction, good carbohydrate content, less antinutrients, fiber	Gluten‐free alternative, new textures & colors, moderate glycemic index	Less firmness, color less appealing, variable consumer acceptance	
Taro baby food/porridge	Taro‐cereal/pulse mixtures, boiled or steamed	Hypoallergenic, high in calcium, potassium, phosphorus, magnesium, vitamins A, C, B, and fiber	Suitable for cereal/lactose allergies, easily digested, slow energy release	Watch for oxalates/phytates, a specific taste sometimes less liked	
Frozen taro	Boil, peel, cut, vacuum pack or bag, freeze	Retains nutritional profile, minimal sensory loss	Enables extended storage (> 6 months), off‐season use, and low cost	Further safety/quality studies needed, potential texture changes upon thaw	
Taro flour as a soup thickener	Drying, milling, and incorporation into soups/sauces	Rich in minerals (ash, calcium), thickener, moderate glycemic index	Diversifies diet, suitable for special diets, easy to use	Less protein and fat than melon or dikanut seeds	
Pounded taro/Achu fufu	Boil corms with skin, peel, and pound in a mortar	Complex carbohydrates, beneficial viscoelasticity, and mucilage dispersal	Popular dish, good texture with yellow sauce, widely accepted	Labor‐intensive, time‐consuming, requires specific equipment, limited availability	
Taro chips/Achicha	Boil, peel, slice, sun‐dry or smoke, optionally fry	Resistant starch, complex carbohydrates, dietary fiber, and extended shelf‐life	Ideal for dry/famine seasons, portable snack	High glycemic index if fried, possible nutrient loss	
Baked/boiled taro leaves	Cook young leaves with coconut or cow's milk, sometimes with other ingredients	High fiber, vitamins A/C, calcium, oxalate are reduced by cooking	Plant‐based nutrition source, oxalate is lowered by boiling/milk	Residual oxalate, kidney stone risk if overconsumed	
Oxisoup (spiced taro soup)	Taro flour, local spices (Dichrostachys, Xylopia, Scorodophloeus), quick broth	High antioxidants (polyphenols/flavonoids), anti‐inflammatory, fiber	Cultural significance, high antioxidant activity, and good sensory acceptance	Spice quality is dependent, needs precise formulation for flavor/health	
Spherical starch aggregates	Extraction and aggregation of taro mucilage/starch, encapsulating ingredients	Food/drug encapsulation, good release capacity, high in amylose and mucilage	Innovative (probiotics, oils, micronutrients), tunable texture	Production cost, access to technology, and needs for industrial validation	
Critical inventory of yam‐based products					
Boiled or roasted yams	Peeling, slicing, boiling in water, or roasting	Carbohydrates, fiber, minerals, and low‐fat	Easy, common, high digestibility	Perishable root, often low in protein	Akinoso and Abiodun (2016)
Yam mash (pounded yams)	Peeling, boiling, pounding, kneading	Carbohydrates, some protein, and fiber	Popular in West Africa, hearty	Very laborious by tradition	
Yam chips	Slicing, blanching, sun or oven drying	Carbohydrates, energy	Long shelf life, traditional snack	Contamination risk, dependent on the weather	
Fried yams/fritters (ojojo)	Peeling, grating, mixing with ingredients, and deep‐frying	Carbohydrates, quick energy, fats	Enjoyed the taste, snack, and convenience	High in fat (fried)	
Stiff yam dough (amala, kokonte)	Sun‐dried yam flour + boiling water, cooked and stirred	Starch, fiber, energy, minerals	Quick prep with instant flour	Texture depends on drying and flour quality	
Instant yam flour	Peeling, washing, cutting, blanching, drying, milling	Mostly carbs, easy storage	Reduces labor, long shelf life	Less traditional, processing cost	
Yam flakes	Steam‐cooked, mashed, drum‐dried, granulated	Ready to use, stable nutritional value	Time‐saving, pleasant taste, easy use	Needs specialized equipment	
Extruded yam products	Yam flour extruded at high temperature for snacks	Rapidly digestible energy, varied uses	High industrial potential, product variety	Tech investment, precise formulation	
Composite flours (bread, cakes)	Yam flour blended with wheat/other flours for bakery	Boost in antioxidants, gluten‐free	Diet diversification is suitable for celiacs	May alter the taste or texture of the product	
Yam starch	Extraction, purification; used as thickener/gelling agent	High in starch, varies by species	Industrial uses (food, pharma)	Underused industrially	
Modified yam starch	Physical, chemical, or enzymatic modification of native starch	Improved viscosity, better texture	Multiple technological uses	Sometimes complex processes	

#### 7.1.1 Cassava Cyanogenic Glycosides: Toxicological Mechanism, Regulatory Limits, and Detoxification Kinetics

Cassava ( *M. esculenta* ) biosynthesizes cyanogenic glucosides, predominantly linamarin [2‐(β‐D‐glucopyranosyloxy)isobutyronitrile] (~95%) and lotaustralin (~5%). When cellular compartments are damaged, linamarin is hydrolyzed by endogenous β‐glucosidase (linamarase) to produce acetone cyanohydrin, which either spontaneously at pH > 5.0 and temperatures > 35°C or enzymatically via hydroxynitrile lyase dissociates into volatile hydrogen cyanide (HCN). In humans, acute ingestion of cyanide (lethal dose 0.5–3.5 mg/kg body weight) arrests cellular respiration by inhibiting mitochondrial cytochrome c oxidase. Chronic low‐level dietary exposure exacerbates iodine deficiency disorders (goiter, cretinism) and induces irreversible motor neuron degeneration known as konzo or tropical ataxic neuropathy (TAN) (Bradbury 2006; Oloya et al. 2023; Borku et al. 2025). To safeguard consumer public health, the Codex Alimentarius Commission established a mandatory maximum limit (ML) of 10 mg/kg (ppm) total hydrocyanic acid in edible cassava flour (CXS 176‐1989) and gari (CXS 151‐1989). Although fresh bitter varieties frequently contain 100–500 mg/kg HCN equivalent, traditional solid‐state and submerged fermentations effectively reduce cyanogenic potential by > 90% through microbial and endogenous linamarase activity, followed by hydraulic dewatering that leaches soluble cyanohydrins, and roasting/frying (e.g., in gari preparation) which drives off residual free HCN below the 10 mg/kg regulatory limit (Sanni et al. 2009; Padonou et al. 2010; Elisabeth et al. 2022). Quantitative monitoring in decentralized production relies on semi‐quantitative picrate paper kits (the Bradbury method) with visual/spectrophotometric colorimetry (510 nm), whereas centralized quality assurance employs automated enzymatic assays and reverse‐phase high‐performance liquid chromatography (RP‐HPLC).

#### 7.1.2 Potato Steroidal Glycoalkaloids: Toxicological Profile, Safety Thresholds, and Processing Behavior

In potato ( *S. tuberosum* ), food safety concerns center on steroidal glycoalkaloids (SGAs), primarily α‐solanine and α‐chaconine, which constitute > 95% of total glycoalkaloid content (TGA). These nitrogen‐containing secondary metabolites exert toxicity through cell membrane lysis via sterol complexation and reversible inhibition of acetylcholinesterase, producing clinical symptoms of nausea, severe diarrhea, hemolytic anemia, and neurological disorientation. A widely accepted international safety guideline established by FAO/WHO and corroborated by the European Food Safety Authority (EFSA CONTAM Panel 2020) defines a maximum threshold of 200 mg/kg fresh weight (20 mg/100 g) TGA in commercial edible potato tubers, with EFSA setting a Lowest Observed Adverse Effect Level (LOAEL) of 1 mg total SGA/kg body weight per day. SGAs are predominantly concentrated in the dermal and sub‐dermal periderm tissues (the outer 1 mm layer), accounting for 80%–90% of total tuber content. Consequently, mechanical peeling achieves a 60%–90% reduction in TGA levels, serving as the most critical primary control point. Because α‐solanine and α‐chaconine are highly thermostable (thermal degradation occurring only above 240°C), conventional boiling and baking confer negligible degradation (< 10%–15%), whereas deep‐frying at 160°C–180°C provides only modest decomposition. Analytical quantification is routinely executed using HPLC coupled with diode array detection (HPLC‐DAD, *λ* = 208 nm) or liquid chromatography–tandem mass spectrometry (LC–MS/MS) (Ji et al. 2012; EFSA 2020).

#### 7.1.3 Taro and Yam Oxalates: Crystallographic Hazards, Mitigation Kinetics, and Analytical Assays

Taro ( *Colocasia esculenta* ) and specific wild yam species (Dioscorea spp.) feature significant concentrations of oxalates, occurring both as water‐soluble oxalic acid and insoluble needle‐like calcium oxalate raphide crystals. Ingestion of raw or insufficiently processed corms induces severe acridity, characterized by painful mechanical micropuncturing of oral, pharyngeal, and esophageal mucous membranes accompanied by histaminic irritation. Systemically, excessive absorption of soluble oxalates sequesters bioavailable calcium and iron, precipitating insoluble calcium oxalate monohydrate in the kidneys and dramatically increasing the risk of nephrolithiasis and renal epithelial damage, particularly in individuals with daily dietary intakes exceeding 200–500 mg/day (Nigussie 2025; Himeda 2012). Unlike potato glycoalkaloids, soluble oxalates exhibit high aqueous solubility; extended hydrothermal boiling (45–60 min) leaches 40%–70% of soluble oxalates into the cooking broth, provided the broth is discarded. Furthermore, prolonged traditional lactic fermentation (e.g., 2–3 weeks for Hawaiian poi or Cameroonian achu preparation) degrades up to 85% of total oxalates through microbial enzymatic breakdown by oxalyl‐CoA decarboxylase and formyl‐CoA transferase pathways present in indigenous Lactobacillus and Bifidobacterium strains (Njintang et al. 2016; Nigussie 2025). In food testing laboratories, oxalate fractions are systematically quantified via reversed‐phase HPLC using UV or refractive index detection, ion chromatography with conductivity detection, and potassium permanganate titrimetry.

### 7.2 Nutritional Complementarity and Biofortification

The nutritional profiles of PTRs are diverse and complementary, dictating their specific roles in the human diet. The sweet potato, particularly orange‐fleshed varieties, is a powerhouse of β‐carotene (provitamin A), making it a critical tool in preventing childhood blindness (Mu, Li, and Sun 2016). Yams and taro provide high dietary fiber, potassium, and antioxidants, supporting digestive health and satiety, whereas cassava's protein deficiency necessitates its pairing with protein‐rich leaves, legumes, or oilseeds (Sharma and Kaushal 2016). Plantain offers a unique profile of gradual energy release, is rich in potassium and magnesium, and is applied across a vast culinary spectrum, from the ceremonial pounded dough (*Ntuba ekōn*) to ubiquitous street foods like alloco (Newilah et al. 2005; Mundéné‐Timothée et al. 2024; Rousselot 2025).

### 7.3 Agro‐Industrial Innovation and Market Expansion

A critical shift is currently occurring where yams and sweet potatoes are moving beyond simple boiling or frying toward sophisticated industrial applications. Innovations such as vacuum‐microwave drying, low‐temperature vacuum frying, and extrusion have enabled the creation of instant noodles, puffed snacks, and even lactose‐free desserts (Kaur and Sandhu 2016; Mu, Sun, and Li 2016). These technologies, as illustrated in the inventory of sweet potato‐based products (Table 6), allow for the production of low‐fat chips and high‐fiber breads that meet the demands of an urbanizing global population. Furthermore, the extraction of high‐quality proteins and pectins from sweet potato residues represents a significant advance in reducing environmental pollution while increasing the added value of the crop (CIRAD 2023).

### 7.4 Socio‐Cultural Significance and Global Integration

Beyond their caloric value, PTRs are deeply embedded in the ritual and symbolic life of societies in Africa, Asia, and Latin America. Yams and taro often embody social cohesion and fertility in festivals, whereas plantain remains a festive staple across the Caribbean and Africa (Akinoso and Abiodun 2016; Adi 2024; Mundéné‐Timothée, Nouga Bissoue, et al. 2025). The rise of “modern‐traditional” products, such as gluten‐free cassava pasta or probiotic‐enriched taro yogurts (Taro Gurt), is disrupting the historical hierarchy that once relegated these crops to “lesser” roles than cereals (Njintang et al. 2016; Dahiya and Nigam 2022).

The future contribution of roots, tubers, and plantains to global food systems depends on the ability of local and industrial processors to harmonize traditional knowledge with modern hygiene and technological mastery. As shown in Table 6, whereas limitations like artisanal inconsistency and high equipment costs remain, the successful integration of these crops into the modern diet offers a resilient pathway toward dietary diversification and sustainable rural development (Obadina and Adekoya 2016; Shitandi and Kihumbu‐Anakalo 2016).

Despite the profound nutritional, culinary, and commercial promise demonstrated by these diverse food applications, scaling them from artisanal kitchen operations to resilient, formalized agro‐industries reveals profound structural bottlenecks. Transforming perishable roots, tubers, and plantains into standardized global commodities exposes systemic vulnerabilities across the value chain, ranging from staggering post‐harvest losses and deficient cold‐chain logistics to gender inequities and fragmented regulatory enforcement, which must be comprehensively resolved.

## 8 Challenges and Prospects in the PTR Value Chain

The transition of PTRs from subsistence staples to high‐value industrial commodities is governed by a complex matrix of interconnected challenges. Addressing post‐harvest losses, neutralizing anti‐nutritional factors, and fostering sustainable production requires a multidimensional approach that transcends simple technological fixes to include infrastructural, financial, and socio‐cultural reforms. As these crops are foundational to the caloric and nutritional security of millions, particularly in Central and West Africa, where plantains alone provide over 25% of the daily intake, the stability of their value chain is a global imperative (Oyeyinka and Afolayan 2019; Eyembe et al. 2024).

### 8.1 Post‐Harvest Losses and Infrastructural Bottlenecks

A primary impediment to global food security is the high rate of post‐harvest losses, which frequently range between 30% and 40% across developing regions, with cassava suffering up to 45% losses during peak harvesting seasons due to rapid postharvest physiological deterioration (PPD) within 24–72 h of harvest and mechanical injuries during unpadded transport (FAO 2019; Hodges et al. 2011; Affognon et al. 2015; Khan et al. 2024; Urugo et al. 2024). PTRs are inherently perishable due to high moisture content and physiological respiration, yet the infrastructure required to mitigate this is often absent. Deficient transport networks and a lack of reliable electricity severely limit the deployment of cold chain technologies (Shapiroe 2024). Although advanced non‐thermal innovations like pulsed electric fields (PEF) show promise in laboratory settings, their industrial scaling is hindered by the high initial costs and maintenance requirements that surpass the capacity of smallholder cooperatives (Urugo et al. 2024). Furthermore, the emergence of phytosanitary threats, such as Fusarium wilt in Latin America, exacerbates production instability, highlighting a critical need for integrated pest management and resilient supply chains (Rocha et al. 2021; Munhoz et al. 2024).

### 8.2 Socio‐Cultural Resistance and Safety Standardization

Mitigating anti‐nutritional factors (ANFs), such as cyanogenic glycosides in cassava and oxalates in yams, faces a “dual barrier” of technical and socio‐cultural constraints. Traditional processing methods are deeply entrenched in local heritage; consequently, newer interventions like enzymatic treatments or biofortified cultivars often encounter skepticism regarding altered taste profiles and unfamiliarity (Urugo et al. 2024). This resistance is compounded by the absence of standardized safety protocols among decentralized, small‐scale producers. Establishing trust through targeted communication and ensuring access to affordable enzymatic inputs are essential steps to harmonize traditional wisdom with modern safety standards.

### 8.3 Gender Dynamics and Socioeconomic Equity in the PTR Value Chain

A defining yet frequently marginalized dimension of global nutritional equity within the PTR sector is the role of gender. Throughout Sub‐Saharan Africa and Latin America, women constitute 60%–85% of the operational labor force engaged in post‐harvest processing, artisanal value addition, and local retail (Forsythe et al. 2020; Teeken et al. 2018; Igboanugo et al. 2024). In the cassava sector, female processors perform virtually all manual peeling, grating, dewatering, and the arduous open‐hearth roasting required for *gari* and *attiéké* production. This labor‐intensive work exposes women to severe occupational health hazards, including chronic respiratory and ocular inflammation from continuous exposure to firewood smoke and polycyclic aromatic hydrocarbons, acute inhalation of volatilizing hydrogen cyanide during grating and frying, and severe musculoskeletal strain from the manual pounding of *fufu*, *achu*, and *ntuba ekōn* (Abiona et al. 2021; Mundéné‐Timothée et al. 2024). Furthermore, socio‐cultural and institutional barriers severely restrict women's access to processing credit, mechanized inputs (e.g., motorized raspers, mechanical hydraulic presses, automated flash dryers), and formal land tenure (Mbudzya et al. 2022; Savoy 2022). Historical evidence indicates that when artisanal food processing undergoes spontaneous mechanization without gender‐sensitive policy interventions, technological control and revenue streams are frequently co‐opted by male entrepreneurs, dispossessing women of their traditional income streams (Forsythe et al. 2020). Consequently, realizing authentic nutritional equity mandates gender‐responsive technology development, targeted microfinance and equipment leasing for female processing cooperatives, and the ergonomic design of clean‐energy processing machinery that eliminates toxic emissions while safeguarding female economic autonomy.

### 8.4 Scalability of Value‐Added Innovations

The transition from artisanal to commercial production of novel products, such as plantain‐based yogurts or cassava‐derived milk analogues, is frequently stalled by technical and market hurdles.

Transitioning to industrial scales demands specialized equipment and expertise that are often unavailable in rural processing hubs (Ansari and Javaid 2024). Moreover, novel food products face intense competition from established global commodities, necessitating significant investment in consumer education and sensory marketing to build brand equity. Financial constraints remain a recurring theme, as small and medium enterprises (SMEs) struggle to access the capital required for high‐grade processing infrastructure and inventory management (Savoy 2022).

### 8.5 Sustainability and Climate Resilience

Promoting agroecological and circular economy models, such as the valorization of processing waste into bio‐energy or animal feed, is often met with behavioral inertia. Smallholder farmers, driven by short‐term economic imperatives, may perceive sustainable practices as labor‐intensive or less profitable in the immediate term (Martinez 2023). This is further complicated by insecure land tenure, which discourages long‐term investments in soil health (Mbudzya et al. 2022). Simultaneously, climate change acts as an intensifying systemic stressor that compounds existing agronomic vulnerabilities and post‐harvest deterioration risks, thereby elevating the risk profile of PTR‐based food systems (Ansari and Javaid 2024).

### 8.6 Strategic Alignment With Sustainable Development Goals (SDGs)

The systemic resolution of these challenges directly aligns with several United Nations Sustainable Development Goals. By improving yields and reducing losses, the sector contributes to SDG 2 (Zero Hunger). The development of biofortified and detoxified products supports SDG 3 (Good Health and Well‐being), integrating female processors into modernized, ergonomic value chains directly advances SDG 5 (Gender Equality), whereas the implementation of circular waste‐to‐value models drives SDG 12 (Responsible Consumption and Production). Achieving these goals requires a multi‐stakeholder platform that integrates policy reform, financial mobilization, and community engagement to bridge the gap between research and field application (Tracex 2024; Urugo et al. 2024). Crucially, a glaring gap in the current PTR landscape is the fragmentation of food safety regulatory oversight. Although international bodies such as the Codex Alimentarius Commission have enacted rigorous quality and safety benchmarks, including maximum limits for hydrocyanic acid in cassava flour (CXS 176‐1989) and gari (CXS 151‐1989), and the European Food Safety Authority (EFSA) provides comprehensive risk assessments on cyanogenic glycosides and glycoalkaloids (EFSA CONTAM Panel 2019, 2020), national enforcement in producing tropical nations remains weak. Regional bodies such as the African Organization for Standardization (ARSO) and national agencies including the Standards Organization of Nigeria (SON, NIS 344/380), Cameroon's Agence des Normes et de la Qualité (ANOR), and the Ghana Standards Authority (GSA) have formulated regional standards. However, because over 80% of PTR commodities circulate within decentralized, informal rural markets, adherence to these standards is rarely monitored or verified. This regulatory disparity leaves domestic consumers vulnerable to toxicological and mycotoxin risks while erecting insurmountable non‐tariff barriers that impede smallholders from accessing lucrative formal export markets.

### 8.7 Future Research Directions

Future investigations must prioritize the synergy between traditional bioprocessing and cutting‐edge technology. Hybrid systems that combine traditional fermentation with PEF could maximize the retention of heat‐sensitive vitamins (C, B9) and polyphenols while simultaneously reducing convective drying duration and thermal energy expenditure by 20% to 30% depending on electric field intensity and matrix porosity (Wiktor et al. 2019; Pateiro et al. 2021; Kumar et al. 2021). On the production side, the use of gene‐editing tools like CRISPR to develop drought‐tolerant cassava (e.g., NextGen Cassava) and yams by modulating stomatal patterning, cuticle wax thickness, and starch branching enzymes (SBE2) can substantially enhance crop water‐use efficiency (WUE) and yield stability under severe drought conditions (Odipio et al. 2020; Luo et al. 2022). Finally, research must be rigorously linked to policy through frameworks like the YIIFSWA initiative, which fosters farmer‐biotechnology‐market interfaces to ensure that technological innovations translate into tangible economic opportunities and national food security (Urugo et al. 2024).

## 9 Conclusion

Plantains, tubers, and roots (PTRs) constitute an indispensable cornerstone of global nutrition, providing a primary energy source for billions, particularly within tropical and subtropical ecosystems. This review has underscored the remarkable versatility of PTRs, tracing their trajectory from foundational traditional staples, such as West African *fufu* and Ivorian *attiéké*, to their emergence as sophisticated vehicles for modern functional foods, including probiotic fermented beverages and gluten‐free pasta.

The integration of major technological advancements marks a pivotal shift in the valorization of these crops. Innovations such as the targeted enzymatic modification of starch allow for the customization of digestibility to meet specific clinical or nutritional requirements. Simultaneously, non‐thermal interventions like high‐pressure processing (HPP) offer a robust mechanism for stabilizing products while preserving volatile, heat‐sensitive vitamins and bioactive compounds. These methods represent a significant leap toward bridging the gap between caloric security and high‐quality nutrient delivery.

However, the transition toward a high‐tech PTR value chain is not without friction. Significant socioeconomic barriers persist, most notably the prohibitive costs of advanced machinery, which limit accessibility for smallholder farmers, the primary producers of these crops. Furthermore, environmental trade‐offs, such as the intensive water consumption characteristic of industrial starch extraction, necessitate a re‐evaluation of current processing paradigms.

For the nutritional and functional potential of PTRs to be fully realized, the research frontier must prioritize a tripartite translational framework:

1. Technological innovation: Leveraging nanotechnology for the encapsulation and controlled release of fragile micronutrients to combat “hidden hunger.”
2. Sustainability: Developing eco‐friendly preservation techniques to drastically reduce post‐harvest losses without escalating the carbon or water footprint.
3. Social inclusion: Ensuring that innovations are scalable and accessible to decentralized processing units to foster rural economic resilience.

Ultimately, PTRs emerge not merely as “undervalued subsistence staples” but as dynamic vectors of agri‐food innovation. By harmonizing technological precision with sustainable practices, gender‐inclusive empowerment, and local knowledge, these crops are uniquely positioned to address the global imperatives of food security, public health, and the United Nations Sustainable Development Goals (SDGs).

## Author Contributions

**Mundéné‐Timothée Junior Lawrence:** writing – original draft, investigation, methodology, software, data curation, visualization, conceptualization, formal analysis. **Veeranna Hitlamani:** visualization, data curation, writing – review and editing, conceptualization. **Njintang Yanou Nicolas:** project administration, writing – review and editing, supervision, conceptualization, validation, resources. **Aashitosh A. Inamdar:** conceptualization, funding acquisition, writing – review and editing, project administration, supervision, resources, validation.

## Funding

The authors have nothing to report.

## Conflicts of Interest

The authors declare no conflicts of interest.

## Data Availability

The data that support the findings of this study are available from the corresponding author upon reasonable request.
